# Non-invasive therapeutics for neurotrauma: a mechanistic overview

**DOI:** 10.3389/fneur.2025.1560777

**Published:** 2025-05-14

**Authors:** James D. O'Leary, Bevan S. Main, Mark P. Burns

**Affiliations:** Laboratory for Brain Injury and Dementia, Department of Neuroscience, Georgetown University Medical Centre, Washington, DC, United States

**Keywords:** traumatic brain injury, plasticity, non-invasive brain stimulation (NIBS), blood brain barrier (BBB), inflammation, transcranial direct current electrical stimulation

## Abstract

Traumatic brain injury is a leading cause of death and a major risk factor for the development of both memory and motor disorders. To date, there are no proven interventions to improve patient outcome after neurotrauma. A promising avenue of treatment has emerged in the use of non-invasive therapies for recovery after brain injury. A number of non-invasive brain stimulation techniques have been developed, such as transcranial direct current stimulation, transcranial magnetic stimulation and vagus nerve stimulation, as well as low intensity ultrasound stimulation and photobiomodulation therapy. However, standardized treatment regimens have not been developed. There is a clear need to better understand the underlying mechanisms of non-invasive therapeutics on brain injury pathology so as to more effectively guide treatment strategy. Here we review the current literature of non-invasive therapies in preclinical neurotrauma and offer insight into the potential mechanism of action and novel targets for the treatment of traumatic brain injury.

## Introduction

Traumatic brain injury (TBI) and stroke are leading causes of death and disability and can lead to the development of a range of cognitive impairments, such as mood and memory disorders (1–3). The complex pathophysiology following neurotrauma make treatment and predicting individual patient prognosis difficult (4, 5). Activity-dependent plasticity of neuronal circuits is considered a key component for successful recovery of function following brain and spinal cord injury (6). Brain plasticity has been a focal point of recovery programs following neurotrauma for many decades. However, no proven interventions have been established to improve patient outcome after brain injury. A promising avenue of treatment has emerged in the use of non-invasive therapies for TBI and stroke (7–9). The ability to non-invasively target key recovery mechanisms, such as enhancing neuroplasticity, reducing inflammation, and attenuating secondary injury cascades, offers a transformative therapeutic approach that avoids the risks and complications associated with invasive interventions. A number of non-invasive brain stimulation techniques have been developed, such as transcranial electrical stimulation (tES), transcranial magnetic stimulation (TMS), vagus nerve stimulation as well as low intensity ultrasound stimulation and photobiomodulation (8). While clinical studies have explored various treatment regimens, outcomes remain inconsistent due to factors such as the heterogeneity of injury, variations in stimulation parameters and the timing of interventions relative to injury onset (10). There is a clear need to better understand the underlying mechanisms of non-invasive therapeutics on TBI and stroke pathology so as to more effectively guide treatment strategy. Preclinical studies have the potential to offer insight into the mechanisms of action of non-invasive therapies, and will lead to the development of more effective clinical treatment. Here we review the current preclinical and clinical literature of non-invasive therapeutics in neurotrauma, focusing on the molecular mechanisms of action and identify key knowledge gaps to guide future research in traumatic brain injury and stroke.

## Traumatic brain injury and stroke

TBI and stroke present significant challenges for patients, caregivers, and healthcare systems worldwide. TBI results from external mechanical forces such as falls, motor vehicle accidents, sports injuries, or military-related incidents, leading to immediate and often progressive brain damage characterized by neuronal death, inflammation, and disruption of the blood-brain barrier (1–3). In contrast, stroke arises from an interruption in cerebral blood flow due to ischemia or hemorrhage, causing localized brain injury and widespread functional deficits (11). Stroke is broadly classified into two main categories: ischemic, caused by thrombotic or embolic obstruction, and haemorrhagic, resulting from vascular rupture (12). The primary treatment strategy for TBI focuses on stabilizing the patient and minimizing secondary injury through interventions such as intracranial pressure management (13). For stroke, treatment emphasizes the rapid restoration of cerebral blood flow, typically achieved through thrombolytic therapy or surgical intervention (11). These acute-phase treatments are critical for improving outcomes; however, they are often inaccessible to patients when injuries occur outside of hospitals or in situations where immediate medical care is unavailable. As a result, many patients miss this vital acute window and instead enter the healthcare system during the late post-injury period, highlighting the need for effective long-term recovery strategies. Post-injury rehabilitation primarily involves physical and speech therapy, which provide sensorimotor input to the central nervous system (CNS), promoting neuroplasticity and CNS remodeling (14). These therapies play a pivotal role in improving functional deficits and enhancing patient quality of life after brain injury. Despite their importance, these programs face several challenges, including slow onset of benefits, high time demands, and low patient compliance (15). Innovative rehabilitation strategies are needed to enhance long-term recovery by targeting neuroplasticity and functional outcomes during the chronic post-injury period. These approaches should complement acute-phase and traditional rehabilitation treatments to improve adherence, recovery, and quality of life for patients with brain injuries like TBI and stroke.

TBI and stroke share pathophysiological mechanisms that exacerbate injury and complicate recovery. Both conditions initiate secondary injury cascades, including excitotoxicity, oxidative stress, inflammation, and apoptosis (16, 17). These processes amplify initial damage and contribute to long-term disabilities. Additionally, alterations in cerebral blood flow, impaired autoregulation, and inadequate oxygenation further compound the effects of the secondary injury, resulting in persistent cognitive deficits, motor impairments, and neurodegeneration (18). There is also an established interaction between these two conditions, where TBI is known to increases stroke risk through vascular injury, blood-brain barrier disruption, and a pro-inflammatory state that predisposes individuals to thromboembolism and hemorrhage (19, 20). Understanding the shared and unique pathophysiological mechanisms of TBI and stroke is essential for developing targeted therapies that improve patient outcomes. Globally, TBI affects an estimated 50–60 million individuals annually, while stroke is the second leading cause of death and a primary cause of disability, impacting approximately 13 million people each year (3, 21). Together, these conditions impose a profound healthcare burden, with global costs exceeding $400 billion annually (3). Despite the significant overlap in the pathophysiology of TBI and stroke, the research fields for these conditions often operate in isolation, with limited cross-disciplinary dialogue. This separation limits opportunities to leverage advancements in one field to inform treatment strategies in the other. Establishing a shared framework for discussing effective and ineffective treatments across these domains could foster collaboration, accelerate progress, and collectively advance therapeutic innovation for both conditions.

## Transcranial electrical stimulation (tES) and brain injury

**Figure 1:**
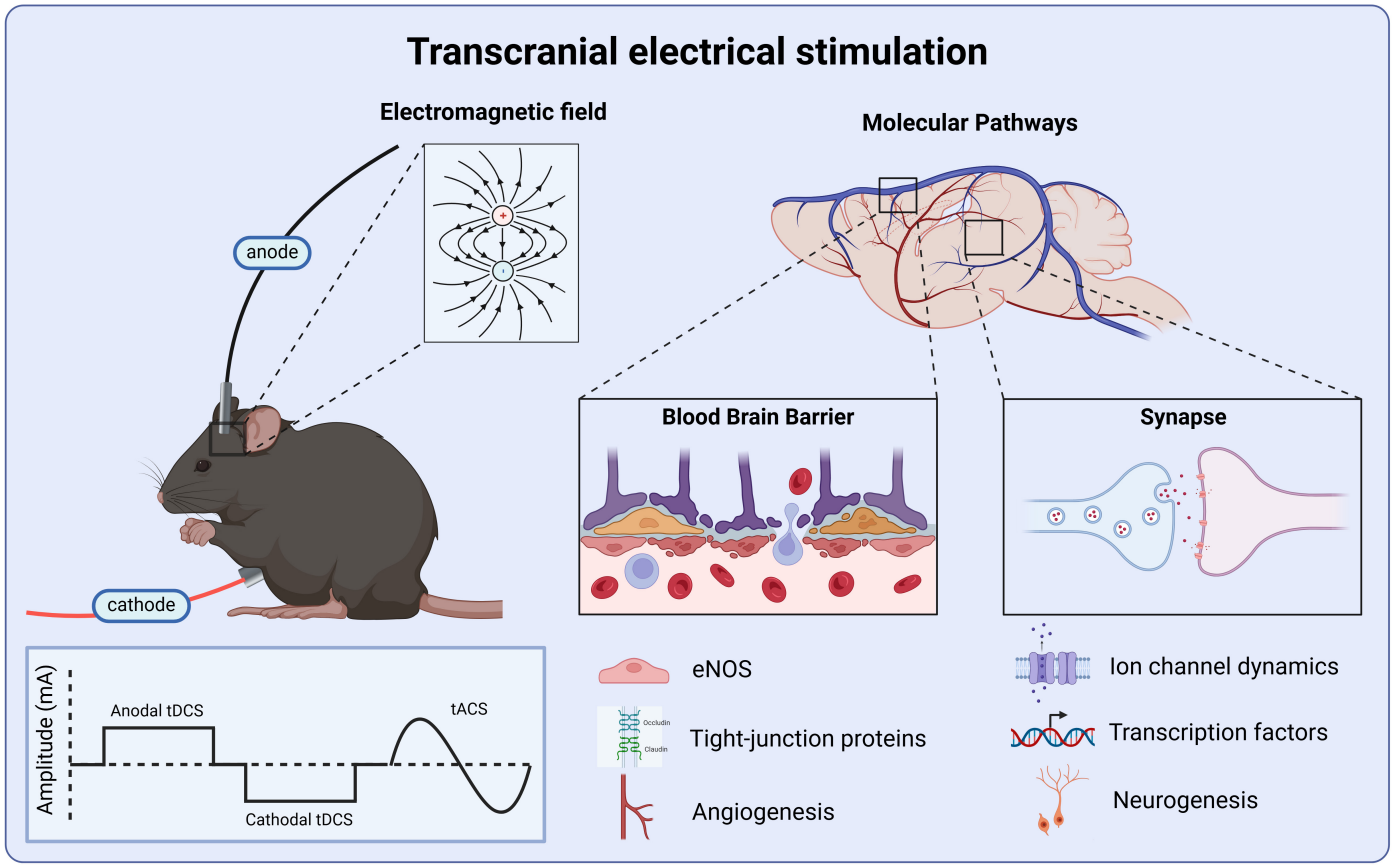
Molecular targets of transcranial electrical stimulation. Anodal and cathodal tDCS deliver a monophasic square waveform, while tACS delivers an alternating sinusoidal wave. All tES protocols produce an electric field which modulates neural activity via the regulation of ionic channel dynamics, leading to transcription changes in cellular signaling, and upregulation of growth factors which promote adult hippocampal neurogenesis and angiogenesis. tES produces anti-inflammatory effects via the up-regulation of eNOS which prevents tight-junction protein breakdown, preserving blood-brain barrier integrity.

Transcranial electrical stimulation (tES) is a non-invasive brain stimulation technique that modulates cortical excitability and promotes neuronal plasticity (Figure 1) (22). It has been employed to treat a wide range of psychiatric and neurological conditions, including TBI and stroke, with promising but variable outcomes (7, 9, 23). In healthy subjects, anodal transcranial direct current stimulation (tDCS) has been shown to enhance motor learning, memory consolidation, and cognitive task performance by strengthening synaptic efficacy and long-term potentiation (LTP)-like processes (24). An intriguing concept involves utilizing tDCS to enhance recovery and rehabilitation by targeting specific cortical regions engaged in task-related learning. For instance, stimulation of Broca's area to facilitate improvements during language tasks (25), while stimulation of the parietal cortex may support learning in visual tasks (26). Similarly, applying tDCS to the primary motor cortex has shown promise in enhancing motor learning tasks (27), and targeting the prefrontal cortex could aid implicit learning and executive function tasks (28). By tailoring stimulation to the neural circuits directly involved in the rehabilitative activity, tDCS may optimize task-specific plasticity and functional recovery.

The primary mechanism by which tDCS exerts its effects is thought to involve changes in membrane polarization, where neuronal depolarization and hyperpolarization alter cortical excitability and synaptic plasticity (29, 30). A key feature of tDCS is its polarity-dependent effects. Anodal stimulation, involving the delivery of a positive current to the cortex, increases neuronal excitability by depolarizing the resting membrane potential (29). This depolarization brings neurons closer to their action potential threshold, enhancing their likelihood of firing in response to subsequent synaptic inputs. The excitatory effects of anodal tDCS are mediated primarily through voltage-gated sodium and calcium channels, which are more readily activated as the membrane potential shifts closer to the threshold (29, 31). In addition, calcium imaging has shown that tDCS induces astrocytic calcium release in the cortex (32). Together, these results show that that tDCS can also induce plasticity through astrocytic Ca2+/IP3 signaling. Conversely, cathodal tDCS, which applies a negative current to the cortex, induces hyperpolarization, moving the resting membrane potential further from the action potential threshold. This hyperpolarization diminishes the likelihood of neuronal firing, effectively dampening cortical activity. The inhibitory effects of cathodal stimulation may involve a reduction in sodium and calcium channel activity, as well as enhanced GABAergic signaling (33, 34). These mechanisms collectively suppress synaptic activity and may contribute to therapeutic benefits in conditions characterized by cortical hyperexcitability, such as epilepsy (35). TBI is believed to induce a chronic state of excitability that increases the risk of epilepsy (36). While anodal tDCS may help reduce inflammation and improve cognitive deficits shortly after injury, cathodal tDCS could be more effective in addressing TBI-induced epilepsy, which tends to develop much later after injury.

Sodium and calcium ion channels play a critical role in TBI and stroke. These signaling pathways mediate excitotoxicity and ionic imbalance during the acute phase, influencing secondary injury cascades and inflammation during the subacute phase, and contributing to long-term maladaptive synaptic remodeling (18). Expression of the voltage-gated sodium channel Na_v_1.3 has been shown to be upregulated following TBI (37). Targeting of the Na_v_1.3 by pharmacological inhibition immediately after a fluid percussion injury significantly reduces edema and neurodegeneration (38). Subsequently, blocking Na_v_1.3 after injury can also lead to improved spatial memory in the Morris water maze (38). Ischemic stroke often leads to a failure in the Na+/K+-ATPase pump, leading to cell necrosis and apoptosis. Ionic homeostasis imbalance has been a focal point of many stroke therapies, targeting multiple ion transporters and channels (39). tDCS-mediated changes to ionic balance, particularly through modulation of calcium and glutamate excitotoxicity, can reduce neuronal damage and improve outcomes following ischemic stroke, emphasizing the therapeutic potential of interventions aimed at restoring ionic homeostasis (40).

A second potential mechanism of tDCS is through the release of neurotransmitters. Research using *in vitro* electrophysiology has demonstrated that an electric field can modify the conductance of voltage-dependent sodium channels within the axon terminals (31). Vasu and colleagues show that a weak electrical field modified the opening and closing dynamics of voltage-dependent sodium channels within the axon terminals (31). These modified sodium channels subsequently amplified the terminal polarization, which lead to presynaptic vesicles release (31). In addition to direct effects on membrane potential, anodal tDCS influences the balance of excitatory and inhibitory neurotransmitter systems. Anodal stimulation enhances glutamatergic signaling, promoting excitatory synaptic transmission, while concurrently reducing GABAergic inhibition in the stimulated region. This shift in excitatory-inhibitory balance not only increases neuronal firing rates but also facilitates functional connectivity between cortical areas, supporting improved task performance and learning. Cathodal stimulation in healthy participants reduces GABA and glutamate, exerting an inhibitory effect (41). Application of a cathodal tDCS protocol in the acute phase of TBI or stroke could limit the release of excitatory neurotransmitters, reducing the excitotoxic damage (42). Indeed, cathodal stimulation applied in the very early stage of experimental ischemic stroke shows a protective effect (43). tDCS could be combined into a multimodal treatment strategy, where cathodal stimulation targets acute injury by inhibiting neuronal activity and preventing excitotoxic damage. In contrast, anodal stimulation could facilitate recovery, enhancing synaptic activity during rehabilitation.

The disruption in neural circuits following traumatic injury often involves the dysregulation of neurotransmitters (44). Cellular death, coupled with neural circuit dysregulation can lead to the excessive release of glutamate following neurotrauma (45, 46). Increased glutamate within the synaptic cleft binds to NMDA receptors leading to an increase in Ca^2+^ and causing the secondary excitotoxic cascade (47). NMDA receptors play a crucial role in normal brain function, including synaptic plasticity and memory formation (48). tDCS modulates NMDA receptor activity, leading to changes in neuronal excitability and plasticity (49). In healthy humans, administration of the NMDA receptor antagonist DMO eliminates the after-effects induced by both anodal and cathodal tDCS, suggesting a role of NMDA signaling in modulating tDCS-induced neuromodulation (29, 33). NMDA receptors are a novel target of neural modulation by tCDS in TBI pathology. However, specific experiments examining the role of NMDA in the mediating the effects of tDCS following traumatic injury are needed. The imbalance of cortical excitation and inhibition, particularly involving glutamate and GABA, is a key factor in the changes in neuronal circuits following TBI (46). The reduction in inhibitory synapses after TBI can contribute to the loss of local inhibition (50). Targeting of the excitatory/inhibitory balance may therefore be a key molecular mechanism of tDCS treatments.

Dopamine is another key neurotransmitter that is disrupted following injury (44). Indeed, TBI has been linked to the development of parkinsonism and increases the risk of Parkinson's disease (PD) (51). PD is characterized by the progressive degeneration of nigrostriatal dopaminergic neurons, which results in significant decreases in tyrosine hydroxylase (TH) expression, a key enzyme in dopamine synthesis. This reduction in TH impairs dopamine neurotransmission (44). Decreased TH expression and dopamine transporter levels in the substantia nigra have been reported following CCI-induced TBI (52, 53). tDCS treatment enhances striatal dopamine release, which leads to improvements in attention and executive function. In healthy patients, tDCS targeting the dorsolateral prefrontal cortex increases dopamine within the right ventral striatum. This increase in dopamine is associated with improved performance in attention and executive function (54). Together, suggesting that tDCS-induced activation of dopaminergic cells may be an underlying mechanism involved in improving cognitive function post-injury (54). Indeed, evidence from pharmacological studies has shown that targeting dopaminergic pathways is a promising therapeutic strategy for managing TBI-related outcomes (55). Treatment of Amantadine hydrochloride, a dopamine reuptake inhibitor, improves cognition function after TBI (55, 56).

The hippocampus has been identified as a region where postnatal neurogenesis continues throughout life (57). These adult born neurons play a critical role in supporting cognitive function, such as learning and memory (58). Neurogenesis has been suggested as a possible therapeutic target for both brain injury as well as other neurological disorders (59). Following traumatic brain injury, there is an acute increase in cell proliferation and a loss of immature neurons (59, 60). This initial increase in cell proliferation ultimately leads to a depletion of the proliferative capacity of the neurogenic niche, contributing to a decrease in hippocampal neurogenesis in the late post-injury phase (61). In addition, the dendritic complexity of newborn neurons is also reduced following TBI (62). This disruption in morphology contributes to malformed synaptic integration of new born neurons, leading to impaired cognition (62). In wild-type mice, neurogenesis within the subventricular zone is upregulated following tDCS treatment (63). Notably, repeated tDCS treatments result in widespread increases in hippocampal neurogenesis. Markers of cell proliferation (Ki67), neuronal differentiation (DCX), and cell survival (BrdU/NeuN) all show significant increases after 10 days of tDCS treatment (64). Furthermore, the ability of tDCS to enhance neurogenesis is mediated through GABAergic signaling, as tDCS treatment reduces GABAergic inhibitory tone (64). Pharmacological restoration of GABAergic inhibition prevents the pro-neurogenic effects of tDCS on adult hippocampal neurogenesis (64). In a rodent stroke model, tDCS administered 3 days after ischemia was shown to induce neurogenesis within the subventricular zone (65). This increase in neurogenesis was associated with an accelerated recovery of motor function (65). Hippocampal neurogenesis plays a pivotal role in maintaining the excitatory and inhibitory balance of neuronal circuits by integrating newly formed neurons into existing networks. Immature neurons, which exhibit heightened excitability due to their lower threshold for synaptic input, contribute to the plasticity and adaptability of neural circuits (66). This increase in excitability temporarily shifts the excitatory-inhibitory balance, facilitating circuit remodeling and supporting learning and memory. Taken together these findings suggest, GABAergic regulation of neurogenesis is a molecular mechanism for tDCS treatment in TBI and stroke pathology.

Enhancing the expression of growth factors represents a promising mechanism by which tDCS promotes neuroplasticity and facilitate recovery (Figure 1). Specifically, tDCS appears to enhance memory and support neuronal plasticity by upregulating growth factors, such as brain-derived neurotrophic factor (BDNF) (67). In a mouse model of stroke tDCS treatment increases neuronal spine density and accelerates motor function recovery (68). These findings identify BDNF-TrkB signaling as a key mediator and molecular mechanism underlying the effects of tDCS on neuroplasticity. Elevated BDNF levels following tDCS treatment correlate with improved spatial memory performance in the Barnes maze (69). Supporting these findings, *ex vivo* slice physiology studies indicate that direct current stimulation augments synaptic plasticity through BDNF and TrkB activation in the mouse motor cortex (70). The ability of growth factors to mediate the effect of non-invasive brain stimulation for injury-related brain pathology is a potential avenue for future investigations. Growth factors act directly upon neurogenesis processes, influencing cell proliferation and fate determination. Increased expression of growth/differentiation factor-5 (GDF5) and platelet-derived growth factor subunit A (PDGFA) is observed following tDCS in a mouse model of ischemic stroke (71). These findings align with earlier studies demonstrating that electromagnetic fields increase gene expression involved in the synthesis of growth factors (72).

Disruption of cerebral blood flow, a hallmark of TBI and stroke, highlights the cerebrovasculature as a promising target for tDCS-based interventions (Figure 1). In a CCI model of TBI, repeated administration of anodal tDCS over 4 weeks increases cortical cerebral blood flow in both injured and sham-control mice (73). Specifically, tDCS enhances arteriolar dilation, increases capillary flow velocity, and improves tissue oxygenation, indicating enhanced microvascular function (73). These cerebrovascular improvements are associated with improved motor performance and spatial working memory (73). Additionally, tDCS enhances cerebrovascular reactivity and improves the regulation of microvessel cerebral blood flow (74). Given the role of nitric oxide in mediating cerebral hemodynamics, endothelial nitric oxide synthase, a critical regulator of cardiovascular function, may serve as a non-neuronal therapeutic target of tDCS (74). Interestingly, mice receiving tDCS 3 weeks post-injury exhibit better recovery compared to those receiving stimulation starting 1 week after TBI, suggesting that the timing of intervention is critical (73). Prophylactic stimulation has also been proposed as a treatment strategy for high-risk individuals. For instance, the application of tDCS prior to injury improves motor and cognitive outcomes (75). This neuroprotective effect is attributed to the regulation of calcium and glutamate levels, which reduces excitotoxicity (75). These findings indicate that microvascular hemodynamic dysfunction represents a viable molecular target for tDCS and highlight its potential efficacy in the late post-injury period (73, 74).

Oscillations in neuronal activity are a fundamental feature of brain function, spanning frequencies from ultra-slow (0.05 Hz) to ultra-fast (500 Hz) (76). These rhythmic patterns of activity regulate cognitive processes, with memory closely linked to theta and gamma rhythms, and attention associated with alpha rhythms (77). Disruptions in neural synchronization are a hallmark of cognitive dysfunctions seen in neurological conditions such as TBI and stroke (78). Sharp-wave ripples (SWRs), high-frequency oscillations crucial for memory consolidation and hippocampal-cortical communication, are particularly susceptible to these disruptions, often correlating with deficits in memory and executive function following brain injury (79). While tDCS has shown promise in modulating neural activity to support recovery, its effects tend to diminish shortly after stimulation (69). This limitation may stem from the monophasic nature of tDCS, which does not fully align with the dynamic oscillatory patterns underlying cognition. Emerging evidence suggests that interventions targeting oscillatory activity, such as transcranial alternating current stimulation (tACS), may help restore these disrupted rhythms and improve functional outcomes (80). tACS offers an enhanced treatment strategy by delivering a sinusoidal current that better interacts with intrinsic neuronal oscillations, providing a more precise tool to influence synchronization and plasticity (81, 82).

The polarity-dependent effects of transcranial electrical stimulation are mediated through complex neurobiological mechanisms, including modulation of membrane potential, ion channel activity, neurotransmitter release, and synaptic plasticity (Figure 1). These mechanisms underpin the potential of tDCS and tACS as transformative tools in neurorehabilitation for TBI and stroke. However, despite the growing clinical adoption of tACS, there is a significant gap in preclinical research comparing its efficacy to tDCS and elucidating their differential impacts on neuronal oscillations and SWRs. Future research must address these gaps by investigating the optimal treatment windows, long-term effects, and washout periods for both tDCS and tACS. By bridging the divide between clinical and preclinical studies, we can more effective establish standardize treatment strategies to enhance recovery after brain injury.

## Transcranial magnetic stimulation (TMS) and brain injury

**Figure 2:**
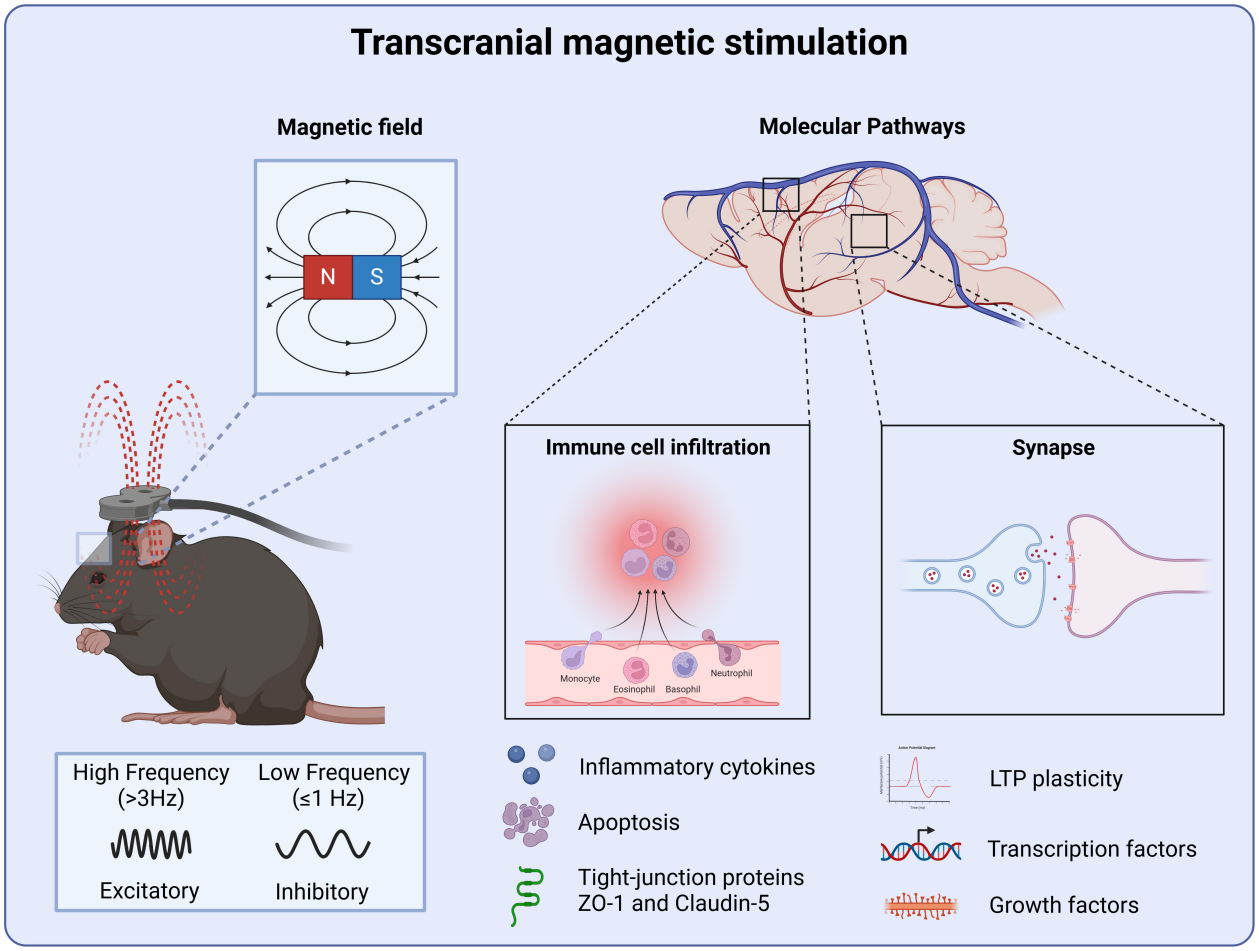
Molecular targets of transcranial magnetic stimulation. TMS produces a magnetic field which is capable of inducing LTP and LTD plasticity. Where high-frequency stimulation produces an excitatory effect and low-frequency stimulation inhibiting neural circuits. Regulating synaptic function through LTP-LTD plasticity leads to the stabilization of neural circuits following injury. TMS also attenuates neuroinflammation and preserve the blood-brain barrier integrity by the reduction of pro-inflammatory cytokines and apoptosis preventing further peripheral immune cell infiltration.

Transcranial magnetic stimulation (TMS) has emerged as a promising therapeutic approach for a number of neurological and psychiatric disorders, including TBI and stroke (83–85). TMS can be thought of as similar to tES, whereby neuromodulation is achieved through the inhibition or excitation of cortical circuits. However, instead of a current passed through the tissue from an anode to cathode or vice versa, a pulsed magnetic field is used to modulate membrane potential (Figure 2) (86). This magnetic field can then either excite or inhibit neuronal tissue depending on the stimulation frequency (87). High-frequency stimulation (>3 Hz) facilitating long-term potentiation, and low-frequency ( ≤ 1 Hz) inducing long-term depression (88, 89). Particular attention has focused on repetitive treatment paradigms, termed repetitive TMS (rTMS). These multiple sessions of rTMS are delivered for several days or weeks (Figure 2) (84, 90). Clinical studies have highlighted the potential for rTMS to facilitate the post-stroke rehabilitation of motor impairment (91), dysphagia (92) and cognitive impairment (93) as well as depression (94). TMS shows promise in aiding recovery from brain injury by enhancing neural plasticity and promoting functional reorganization. However, although both human and animal studies have shown promising results, the specific therapeutic target of TMS remains unclear.

Brain injury is often characterized by dysfunction in monoaminergic and glutamatergic neurotransmitter systems, suggesting that the therapeutic benefits of rTMS may arise from its ability to regulate these critical signaling pathways. Glutamate-mediated excitotoxicity is a well-established mechanism in the secondary injury cascade following brain injuries, leading to neuronal damage and disrupting the balance between excitatory and inhibitory neurotransmission (95). Preclinical studies support the role of rTMS in modulating neurotransmitter activity: in wild-type mice, high-frequency rTMS delivered over multiple sessions upregulates mRNA expression of glutamate and GABA transporters, such as EAAT4, GLAST, GLT-1, GAT2, and GAT4, within the cerebellum, suggesting a restoration of excitatory-inhibitory balance (96). Similarly, rTMS targeting the frontal or caudal cortex in rats has been shown to elevate dopamine and glutamate levels in the nucleus accumbens, further demonstrating its potential to regulate key neurotransmitter systems (97). In neurodegenerative diseases such as Alzheimer's, where neurotransmitter dysfunction is a hallmark of the disease, rTMS has demonstrated its ability to enhance cholinergic activity, vital for learning and memory. Specifically, TMS targeting the prefrontal cortex and hippocampal networks improves cholinergic signaling and boosts brain-derived neurotrophic factor (BDNF) levels, as observed in the 3xTgAD mouse model of Alzheimer's disease (98). These changes correspond to improved cognitive performance and highlight the therapeutic potential of TMS in targeting hippocampal and cortical regions (98). Similarly, in Parkinson's disease, TMS improves motor symptoms, possibly by increasing dopamine release in the basal ganglia (99). These neurotransmitter-driven mechanisms suggest that TMS could facilitate recovery in brain injury by restoring balance in disrupted neurochemical systems.

LTP represents a key mechanism by which TMS-induced neurotransmitter modulation translates into sustained therapeutic benefits. By enhancing the release of neurotransmitters such as glutamate and modulating receptor activity, TMS not only restores excitatory-inhibitory balance but also creates an environment conducive to LTP. This process enables the strengthening of synaptic connections, which is critical for recovery in conditions such as TBI, stroke, and neurodegenerative diseases. During the post injury period the hippocampus undergoes atrophy and exhibits chronic LTP deficits (100). Disruption to hippocampal LTP and LTD contribute to the development of cognitive deficits observed following TBI (101). Ca^2+/^calmodulin-dependent kinase II (CaMKII), a gene known to be involved in LTP, has been shown to be decreased following brain injury (90). Interestingly, administration of TMS for 4 weeks has been shown to be sufficient to restore CaMKII expression to control levels in a CCI model of TBI (90). These findings suggest that repeated TMS acts to promote synaptic plasticity by stabilization of LTP and LTD through Ca^2+^ ion channel dynamics. It is possible that TMS improves recovery following TBI through the induction of LTP-like plasticity, and thereby overcoming injury-induced deficits in hippocampal LTP. Understanding how TMS drives LTP provides valuable insights into its potential to facilitate lasting neurorehabilitation.

TMS enhances the release of neurotransmitters such as glutamate and modulates receptor activity, restoring excitatory-inhibitory balance but also fostering an environment conducive to LTP. After brain injury, the hippocampus undergoes atrophy and exhibits chronic deficits in LTP, which contribute to cognitive impairments (100, 101). Disruption of hippocampal LTP and LTD following TBI has been linked to reduced expression of the synaptic plasticity gene CaMKII (90). TMS administered over 4 weeks restored CaMKII expression following TBI, suggesting its potential to stabilize LTP and LTD through modulation of Ca2+ ion channel dynamics (90). This enhancement of synaptic connections is essential for brain injury recovery, and the ability of repeated TMS to promote synaptic plasticity and stabilization could play a significant role in long-term recovery outcomes.

Alongside modulating neurotransmitter signaling, TMS has been shown to influence neurotrophic growth factors. Including BDNF, which has been hypothesized as being a key player in mediating the efficacy of rTMS (102). Indeed, repetitive TMS has been shown to enhance cortical plasticity in the prefrontal cortex through BDNF-TrkB signaling as well as TrkB-NMDA receptor interaction (103). The extracellular signal-regulated kinase (ERK2) and phosphoinositide 3-kinase (PI-3K) activity in the prefrontal cortex increases following TMS (103). In the context of brain injuries, TrkB signaling facilitates the functional recovery after traumatic injury (104), however this does not occur in anesthetized rats where high-frequency TMS stimulation decreases BDNF and GluR1 (105). These findings demonstrate that the brain state, i.e., wakefulness, has a crucial effect on lasting outcomes of rTMS treatment. This is an important point for future studies, as it is often technically difficult to administer TMS or tES in freely moving animals and therefore many studies perform the stimulation under anesthesia. The development of customized devices capable of enabling non-invasive stimulation in awake, behaving animals will serve as a valuable tool for future preclinical research. Indeed, most commercially available TMS coils are designed for clinical use in humans and are then adapted for small animal studies (106). Although, the development of rodent equivalent rTMS devices poses many technical challenges, such as overheating and coil rupture (107). Several studies have made progress in developing miniaturized setups to allow for improved focal stimulation in rodents (107–109).

TMS also attenuates neuroinflammation. In a weight drop model of TBI, rTMS delivered over 4 days was sufficient to decrease glial fibrillary acidic protein (GFAP) levels in the hippocampus, a marker of astrocyte injury (110). A behavioral recovery was also observed in the same animals, with improvements in motor learning and object recognition (110). In a hemicerebellectomy model of TBI, rTMS was shown to reduce both apoptotic cell death and inflammation within the cortex (111). The reduction in neuronal death was mediated by blocking cytochrome-c release, a key regulator of apoptosis (111). Interestingly, both astrocyte and microglial activation was reduced up to 1 month post injury, suggesting a prolonged anti-inflammatory effect of rTMS in TBI (111). In a similar injury related study, neuropathic pain was modeled by chronic constriction of the sciatic nerve (112). TMS delivered for 14 days restored the anti-inflammatory cytokine IL-10 to control levels (112). Theta-burst TMS has also been shown to ameliorate the infiltration of peripheral immune cells and reduce pro-inflammatory cytokines in a rodent stroke model (113). Theta-burst TMS improved microvascular perfusion and neovascularization (113). Blood-brain barrier permeability was preserved through increased expression of scaffold and tight junction-associated proteins including Zonula Occludens-1 and Claudin-5, which are key components that regulate blood-brain barrier integrity following TBI (113).

Both clinical and preclinical studies underscore TMS as a promising therapeutic tool for promoting recovery after TBI and stroke. Preclinical evidence suggests that TMS exerts its primary therapeutic effects by regulating neuronal activity fostering LTP and LTD plasticity, and stabilizing neural circuits disrupted by injury. In the early post-injury period, TMS mitigates secondary damage by reducing neuroinflammation and preserving blood-brain barrier integrity, thereby preventing peripheral immune cell infiltration and protecting against further inflammatory insults (Figure 2). Furthermore, the ability of TMS to target long-term synaptic plasticity highlights its potential to address chronic motor and cognitive impairments that often persist well beyond the acute phase. These findings collectively position TMS as a versatile intervention capable of supporting both early stabilization and long-term rehabilitation in brain injury recovery.

## Low intensity ultrasound stimulation and brain injury

**Figure 3:**
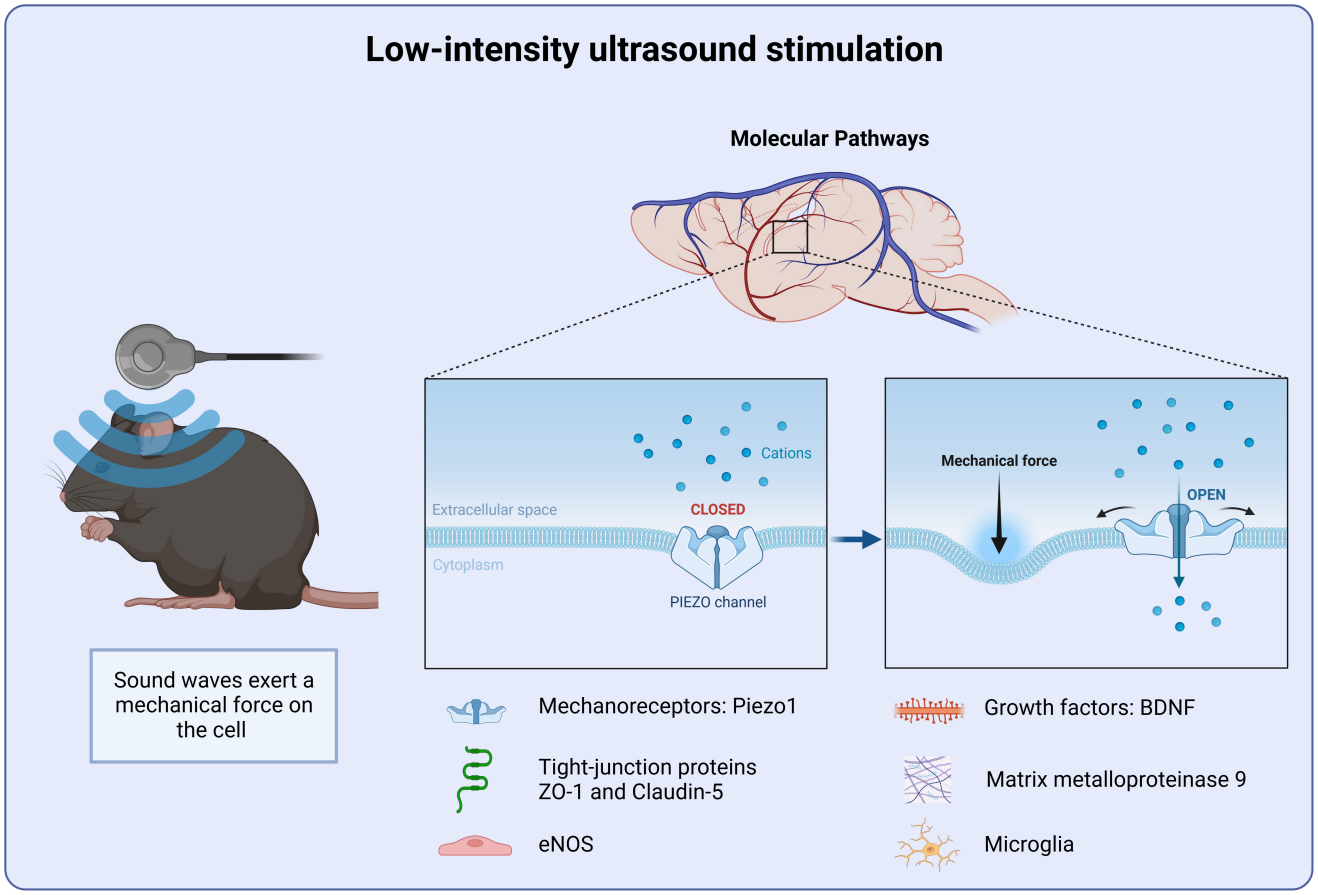
Molecular targets of Low-intensity ultrasound stimulation. Low intensity ultrasound stimulation targets the mechanoreceptor Piezo1. Piezo1 detects changes in the mechanical force of the cellular microenvironment. Piezo1 helps to regulate intracellular calcium homeostasis and preventing further inflammatory signal cascade.

Low-intensity ultrasound has been proposed as an avenue for the treatment of CNS disorders, for review see (114). Low-intensity ultrasound is fundamentally different from the other neuromodulation techniques previously discussed, which utilized electrical currents to generate an electric or magnetic field. Ultrasonic stimulation involves the application of sound waves to generate mechanical forces to effect cellular and molecular structures within the targeted tissue. The non-invasive nature and potential for targeted delivery make ultrasound stimulation a promising avenue for TBI treatment. Ultrasound produces a sound wave that causes mechanical vibrations that passes through the tissue. These vibrations can stimulate mechanosensitive ion channels within neurons (Figure 3) (115, 116). The mechanosensitive cation channel Piezo1 is a key regulator of ultrasonic stimulation of neuronal activity (117), and is functionally expressed in different brain regions and CNS cell types (118). Mechanistically, low-intensity low-frequency ultrasound has been shown to activate Piezo1, which in turn increases Ca^2+^ influx and up regulates c-Fos expression and neuronal activity (117). Piezo1 is gated by membrane curvature and therefore changes in the membrane structure activate this ion channel (119, 120). Interestingly, recent studies have shown that Piezo1 plays a key role in mediating hyperemic CNS blood flow, which is critical for maintaining brain health. Activation of Piezo1 initiates a mechano-feedback mechanism that facilitates blood flow recovery, while endothelial-specific genetic modifications of Piezo1 lead to impairments in complementary memory tasks (121). Furthermore, Piezo1 has been identified as essential for the proper development and function of meningeal lymphatic vessels. Transgenic activation of Piezo1 has been demonstrated to enhance cerebrospinal fluid (CSF) outflow by improving lymphatic absorption and transport (122). These findings suggest that non-invasive ultrasound could enhance Piezo1-mediated lymphatic and glymphatic flow, potentially aiding in the removal of waste proteins. This represents an area of research that remains unexplored in the context of TBI. Indeed Amyloid β (Aβ) peptides are associated with the development of TBI induced cognitive impairments (123), and their formation can disrupt membrane mechanics (124, 125). Furthermore, Aβ monomers have been shown to inhibit Piezo1 function (126). Together, these findings suggest that Piezo1-Aβ interaction may also be a molecular target of the low-intensity ultrasound stimulation in TBI (Figure 3). Mechanoreceptor receptor activity within the spinal cord and brainstem also play a key role in the neuronal representation of touch (127, 128). Targeted up regulation of mechanoreceptors within the spinal cord and brain stem may also be a potential avenue to treat the somatosensory impairments caused by traumatic injury.

In addition to its effects on mechanoreceptors, ultrasonic stimulation ameliorates the post injury inflammatory environment. Indeed, the majority of TBI preclinical studies have focused on the anti-inflammatory effects of ultrasound stimulation. As previously discussed, the blood-brain barrier is known to be disrupted after brain injuries (129). Tight junction proteins such as claudin and occludin degrade, causing a break down in the blood-brain barrier integrity (130). This increase in permeability then leads to an increase in cerebral edema. Cerebral edema is a major contributing factor for patient outcome following brain injury (130). In a rodent model of blood-brain barrier disruption, ultrasound stimulation significantly reduced the level of brain edema, as well as neuronal death and apoptosis (131). A similar result was also observed in a CCI model of TBI (132). Ultrasonic stimulation led to a reduction in cerebral edema and a decrease in neutrophil infiltration as well as microglia and MMP-9 activity (132). This finding was support by another study using a weight-drop model of TBI, where Evans blue absorption was significantly reduced 5 h post injury in mice treated with ultrasound (133). Together, indicating that the ultrasonic stimulation reduced the degree of blood-brain barrier disruption after injury. These neuroprotective effects have also been observed up to 1 month post injury in a CCI model of TBI (134). Ultrasound stimulation may also attenuate inflammation through the regulation of cytokine signaling. Low-intensity ultrasound has been shown to promote OX-A/NF-κB/NLRP3 signaling pathway (135). Repetitive stimulation with low-intensity ultrasound *in vitro* has also been shown to modulate GABA levels via TRPA1, which is expressed in astrocytes (136). Together these finding suggest that ultrasound stimulation may regulate neuronal function and recovery via astrocyte-neuronal interactions.

Ultrasonic stimulation can also enhance cell proliferation and differentiation, as well as protein synthesis. Low-intensity ultrasound has been shown to promote the release and upregulation of growth factors, such as BDNF and VEGF (137). In addition, it has been demonstrated to enhance the phosphorylation of TrkB, Akt and CREB. Interestingly, this effect was inhibited when BDNF signaling was down regulated (137). *In vitro*, ultrasound has been shown to enhance the expression of BDNF in astrocyte culture, through activation of TrkB-Akt and calcium-CaMK signaling pathways (138). Suggesting BDNF as a key regulator of ultrasonic stimulation. Upregulation of growth factors promote non-neuronal cell growth. Ultrasound stimulation has been shown to facilitate the production of pro-angiogenic factors. Low intensity ultrasound promotes the formation of new blood vessels, which in turn improves local blood supply and oxygenation, facilitating recovery after injury. Low intensity ultrasound stimulation targets multiple neuroprotective mechanisms, such as reducing pro-inflammatory cytokines and preserving the blood-brain barrier integrity as well as the up-regulation of growth factors (Figure 3).

## Transcranial photobiomodulation and brain injury

**Figure 4:**
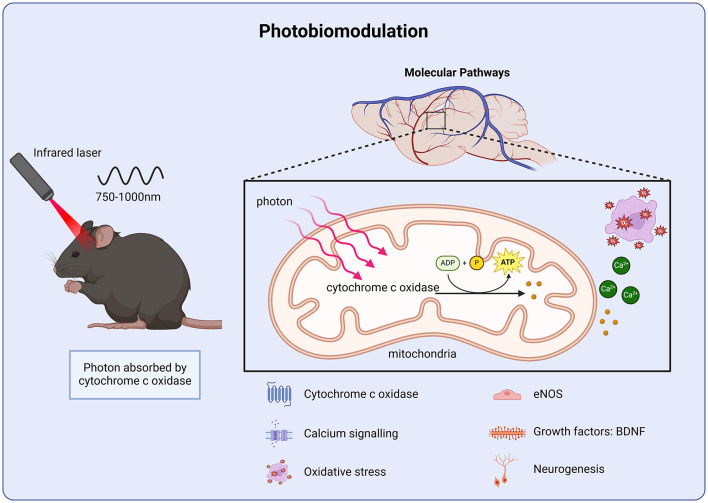
Molecular targets of photobiomodulation. Photobiomodulation enhances ATP production via cytochrome c activity in the mitochondrial membrane, improving calcium homeostasis and protecting against oxidative stress as well as stimulating growth factor transcription and cell proliferation.

Transcranial near-infrared light has been proposed as a treatment of neurodegenerative diseases, including TBI (139). Transcranial photobiomodulation, low-level or low-energy laser therapy utilizes far-red and near-infrared light (600–1,400 nm) to penetrate tissue to effect cellular processes (140). The primary mechanism of action involves photon absorption by cytochrome c oxidase within the mitochondria (Figure 4). This photon absorption leads to an increase in ATP production and subsequent up regulation of transcription factors (140, 141). The increase in cellular activity contributes to improved metabolic functioning (142, 143). CCI-induced injury disrupts Ca^2+^ homeostasis, leading to an increase in Ca^2+^ absorption within the mitochondrial membrane (144). The increase in Ca^2+^ ion uptake inhibits respiratory chain-linked electron transfer and energy transduction (Figure 4) (144). These findings are in agreement with previous work that pharmacologically targeted mitochondrial dysfunction after TBI. Administration of a mitochondrial uncoupling prodrug 24 h after a CCI-induced injury, improved mitochondrial bioenergetics as well as reducing oxidative stress markers (145). In addition, low-energy laser irradiation has been reported to reduce scar formation in a rodent model of myocardial infarction (146). Interestingly these results correlated with an increase in ATP levels within the ischemic area. It was suggested that the laser-irradiated myocardial cells may experience a slower rate of injury induced degeneration due to an increase in ATP production (146). Together these studies suggest that photobiomodulation improves TBI recovery by facilitating mitochondrial function. Understanding the relationship between injury induced changes in metabolism and functional outcomes is essential for the development of effective therapies for TBI (147).

The balance between endogenous pro-oxidative and anti-oxidative enzymes is critical for many key cellular processes (148). Oxidative stress occurs when this balance in disrupted and excessive levels of pro-oxidative enzymes are produced. Oxidative stress plays a critical role in brain injury pathology (149, 150). In the case of TBI, there is an excessive production of reactive oxygen species (ROS). The increase in pro-oxidative enzymes significantly contributes to the secondary injury cascade (151). The overproduction of ROS causes damage to DNA as well as lipid peroxidation and protein oxidation (152). ROS can downregulate scaffold and tight junction-associated proteins, such as ZO-1 and claudin-5 (130, 153). The protein oxidation of ZO-1 and claudin-5 leads to an increase in membrane permeability, resulting in an increase in cerebral edema and the infiltration of peripheral immune cells (154). Low-level laser therapy has been shown to reduce oxidative stress in cortical neuronal cultures (155). Huang and colleagues demonstrated that 810 nm laser suppressed ROS production induced by oxidative stress and rescued primary cortical neurons from apoptosis (155). Nitric oxide is a key regulator of vasodilation (156). Endothelial nitric oxide synthase (eNOS) is essential for maintaining vascular function (156). Disruption to eNOS due to brain injuries contributes to impaired cerebral blood flow and oxygenation (157). Photobiomodulation has also been shown to increase nitric oxide production (158). The up regulation of nitric oxide following injury helps to improve cerebral blood flow and oxygenation. Light-induced increase in mitochondria ATP coupled with a reduction in reactive oxygen species protect endothelial cells within the microvasculature from further injury. The therapeutic effect of low-level laser therapy also extends beyond metabolic processes. Near-infrared light can stimulate neurogenesis (139, 159). Xuan and colleagues reported a dose-dependent effect of laser stimulation on cell proliferation (160). Administration of a low-level laser stimulation for 3 days after CCI-induced injury resulted in an increase in cell proliferation, whereas stimulation for 14 days showed a similar level of proliferation as control mice (160). In a subsequent study, BDNF, a key regulator of neurogenesis was also increased following laser stimulation. (161). Previous studies have utilized primary observation tests, such as the Neurological Severity Score, to measure functional outcomes (162–164). Indeed, photobiomodulation administered after TBI has been shown to improve Neurological Severity Scores, suggesting improved recovery (162–164). However, detailed experiments that investigate specific neural circuits involved in complex behaviors, such as learning and memory, executive function as well as emotional regulation are needed.

## Vagus nerve stimulation (VNS) and brain injury

**Figure 5:**
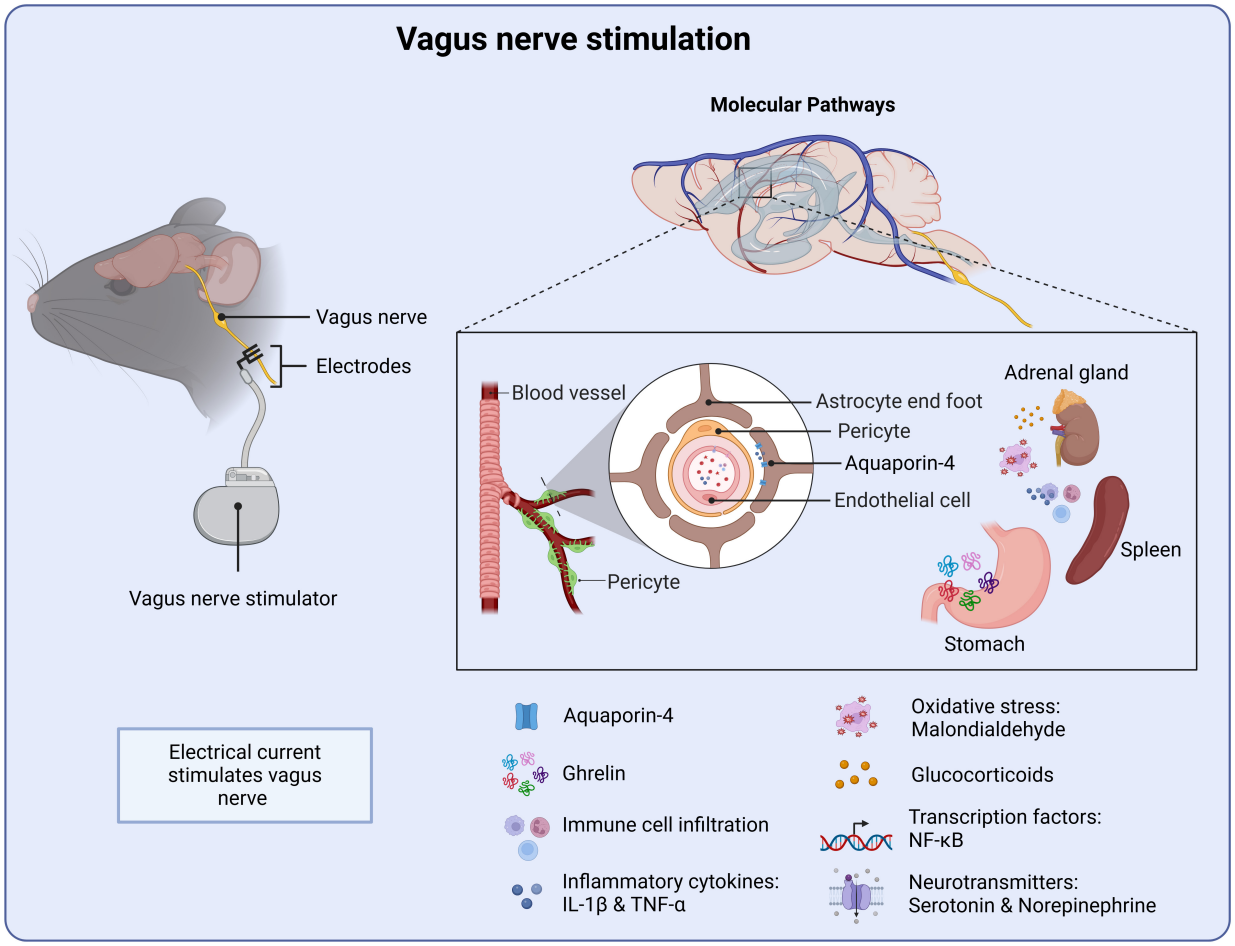
Molecular targets of vagus nerve stimulation. VNS stimulation initiates several anti-inflammatory processes from the periphery. Targeting the water channel Aquaroin-4 as well as reducing pro-inflammatory cytokines and oxidative stress. VNS also stimulates the release of the gastrointestinal peptide ghrelin, which supports neuronal function and recovery.

The vagus nerve is a major component of the parasympathetic nervous system and is involved in the innervation of multiple organ systems. It has been shown to play a key role in the immune response, as well as a mediator of the gut-brain-axis (165, 166). The vagus nerve has therefore become the focus of many treatment strategies for a number of disorders, including brain injuries (167, 168). Vagus nerve stimulation (VNS) is distinct from the other types of non-invasive treatments as the primary mechanism of action is electrical stimulation of the peripheral nervous system, whereas tDCS, TMS and ultrasound as well as photobiomodulation have directly targeted the brain (Figure 5). VNS has shown promise in improving cognitive function and reducing neuropathology in animal models of brain injury and stroke (169, 170). However, the specific mechanism remains to be fully understood.

Oxidative stress plays a key role following brain injury and therefore serves as a therapeutic target of VNS. Tang and colleagues, administered VNS 30 min after injury, in a weight drop model of TBI. VNS was shown to reduce tissue damage as well as brain water content (171). Similar findings were reported when VNS was administered 2 h or 24 h after injury (172). VNS delivered during the early post-injury period and continued for 14 days was also shown to facilitate behavioral recovery, with improvements in motor learning and hippocampal-dependent spatial memory (173, 174). Malondialdehyde, a marker of oxidative stress, is known be elevated following TBI (171). However, mice treated with VNS displayed significantly reduced levels of malondialdehyde, suggesting a reduction in oxidative stress. Furthermore, VNS reduced expression of NF-κB, a key regulator of inflammation, in both the cytoplasm and nucleus (171). In addition, the nucleotide-binding domain (NOD)-like receptor protein 3 (NLRP3) was also down-regulated. Expression of apoptosis-associated speck-like protein (ASC) and caspase-1 was also significantly downregulated by VNS (171). Together, these findings suggest that a key target of VNS is the down-regulation of pro-inflammatory signaling pathways (Figure 5).

VNS may also target endothelial cell function. VNS has also been shown to attenuate cerebral vascular permeability and decrease the up-regulation of aquapoirn-4 after injury, in a weight drop model of TBI. Aquapoirn-4 (AQP4) is known to be upregulated following neurotrauma and has been linked to p-tau accumulation (175). Moreover, previous studies have suggested dysregulation of AQP4 as a contributing factor for cognitive impairments following TBI (175). Injury-induced dysfunction of blood-brain barrier permeability would allow albumin and other plasma proteins into the CNS, thereby directly impacting the inflammatory response by further activating astrocytes and microglia (Figure 5) (176).

Both the blood-brain barrier and the blood-cerebrospinal fluid barrier are ideally positioned to mediate vagus nerve-gut microbial signals, constituting a gateway for gut-brain communication (165). Another potential mechanistic pathway of VNS is through the gastrointestinal peptide ghrelin (Figure 5). VNS has been shown to influence the regulation of ghrelin, which plays a key role in appetite control, metabolism and neuroprotection (177). Modulating vagal afferent pathways can alter ghrelin secretion, impacting metabolism as well as cognitive function through ghrelin's effects on brain circuits involved in neuroplasticity and mood regulation (178). Ghrelin has been demonstrated to exert neuroprotective effects following TBI and ischemic stroke (179). It has been demonstrated to improve neuronal cell survival, memory deficits, and reduce brain injury and functional recovery after TBI and hemorrhagic shock (180, 181). More specifically, ghrelin has been shown to attenuate intracerebral hemorrhage by inhibiting NLRP3 inflammasome activation and promoting the Nrf2/ARE signaling, a key regulator of oxidative stress (182). Pro-inflammatory cytokines, such as IL-1β and TNF-α are significantly elevated following TBI (176, 183). Indeed, these cytokines form the basis of the injury-induced inflammatory response and as such have been the focus of many treatment strategies. Previous work has shown that treatment of VNS following brain injury prevents the injury-induced increase of TNF-α, in a weight drop mouse model of TBI (184). This neuroprotective effect was mediated through ghrelin, where VNS increased serum ghrelin and decreased TNF-α following TBI. Indeed, administration of a ghrelin receptor agonist attenuated the neuroprotective effects of VNS on TNF-α (185). Taken together, these findings suggest that ghrelin is a promising therapeutic target of VNS intervention for TBI as well as other brain injuries.

Long-term loss of arm function is a common motor deficit after ischemic stroke, and significantly contribute to reduced quality of life. Both preclinical and clinical studies demonstrate that VNS combined with rehabilitation training improve recovery of motor function after ischemic stroke (186). In a study conducted by Pruitt et al., VNS was paired with rehabilitation following TBI in a CCI model (187). VNS paired with physical training over a period of 5 weeks significantly increased recovery of both forelimb strength and success rate on the isometric pull task compared with rehabilitative training without VNS (187). In a rodent model of stoke, VNS enhances plasticity in the corticospinal motor networks, leading to increases in synaptic connectivity to musculature of the rehabilitated forelimb. Importantly, the function benefits generalized to another untrained motor task. In addition, the therapeutic effect persisted up to a month after treatment cessation (188). In a clinical study, patients with arm weakness 9 months after ischemic stroke received a rehabilitation program combined with vagus nerve stimulation (189). VNS administered with rehabilitation training improves motor impairment and function compared to only physical therapy (189). It is possible that VNS-induced plasticity is regulated by norepinephrine and serotoninergic signaling. 190 showed that both norepinephrine and serotonin were required for VNS-dependent enhancement of motor cortex plasticity (190). Noradrenergic signaling has also been implicated as a potential mechanisms by which VNS attenuates injury recovery (191). These findings highlight the significance of combing targeted plasticity enhancing therapy, such as VNS or indeed other forms of stimulation such as tES, with rehabilitation programs to improve chronic motor deficits, such as upper limb impairments after TBI or stroke.

## Conclusions and future directions

The complex pathophysiology following TBI and stroke make treatment and predicting individual patient prognosis difficult (4, 5). However, the development of non-invasive therapies offers a path toward rehabilitation. These techniques are safe and simple to administer with relatively little to no adverse side effects (7–9). However, the field of non-invasive brain therapy is still in its infancy with many fundamental questions remaining unanswered. We propose a number of avenues for future investigations.

## Acute and chronic post-injury treatment

The timing of therapeutic intervention may determine the most effective molecular target. Generally speaking, treatment strategies for brain injuries can be divided into two phases. The first focusing on the early post-injury period, which targets apoptosis and edema to ameliorate inflammation and prevent the second injury cascade. The second focuses on the synaptic dysfunction and the ensuring cognitive deficits that emerge in the late post-injury period. While many non-invasive stimulation techniques, such as TMS and tES have demonstrated benefits during the acute and chronic phases, it remains unclear whether certain techniques are more effective when applied at specific time points. Future studies could directly compare different stimulation modalities administered during the same post-injury periods. For example, experiments could assess whether early administration of TMS, tES or Ultrasonic stimulation better reduces edema and neuroinflammation. Additionally, these same stimulations could be administered during the chronic injury phase their effects on restoring synaptic plasticity and cognitive performance compared. Such comparative studies would be instrumental in identifying optimal interventions parameters. In addition, the targeting of eNOS and AQP4 during the early post injury period may provide both a protective mechanisms of blood-brain barrier integrity as well as enhancing the capacity of fluid clearance which may prevent a runaway injury cascade and immune cell infiltration. Future studies could determine the effective time window for targeting eNOS and AQP4 post on recovery outcomes at later post injury time points. It is likely that the therapeutic effect of interventions which target the blood-brain barrier follow the natural injury time course, where protection and support of the blood-brain barrier is most effective before its functional capacity is over run by an injury cascade. Future experiments are needed to characterize blood-brain barrier repair following non-invasive stimulation throughout the acute and chronic injury period.

## Treatment duration

Little is known about the specific molecular pathways that mediate the therapeutic effects of non-invasive brain stimulation, or how long treatment should be continued to achieve lasting benefits. Determining how to sustain or prolong these effects is critical. In the case of treatment-resistant depression, it has been suggested that increasing the number of rTMS sessions can lead to improved outcomes (192). Future studies should investigate whether similar principles apply in the context of brain injury. Specifically, research could explore how varying the duration and frequency of stimulation promotes recovery and whether these parameters are more beneficial for reducing inflammation or enhancing cognitive function. Given the heterogenous nature of brain injury, a universal optimal stimulation parameters may not exist. Indeed, the most effective stimulation parameters will likely be determined by the brain region that is targeted for recovery. Future work could determine stimulation parameters for different brain regions, such as prefrontal compared to temporal cortex. The potential to combine non-invasive stimulation with pharmacological interventions to enhance efficacy is a relatively unexplored area. There is a clear need to better understand the underlying mechanisms through which non-invasive therapies influence TBI and stroke pathology, in order to more effectively guide clinical applications.

## Neuroimmune interactions as therapeutic targets

It is well established that brain injuries disrupt typical neuroimmune interactions (193). Indeed, our understanding of the contribution and crosstalk between the CNS and peripheral immune cells following TBI has increased in recent years (194). Of particular focus is the role of the meninges and the glymphatic/lymphatic system in neurotrauma. Not only do the meninges harbor immune cells that contribute to a vast array of responses, the meningeal lymphatic vessels (mLVs) that reside within them play a crucial role as the waste-removal system in draining CSF, ISF, and CNS-derived molecules from the brain parenchyma to the deep cervical lymph nodes (dCLNs) (194). TBI damages mLVs up to 2-months post injury, and influences adaptive immune responses at the meningeal interface (195–197). However, stratification of the level of damage to the LVs, subsequent lymphangiogenic recovery, and the impact of CSF flow and LV drainage on the removal of proteins such as Aβ after injury remain to be fully characterized. Even less is known about how non-invasive transcranial therapies, whose therapeutic action must pass through the anatomical structure of meninges, influence glymphatic and neuroinflammatory responses after injury. Future research could investigate how meningeal immune cells respond to non-invasive stimulation. Moreover, what stimulation modality best facilitates meningeal and lymphangiogenic recovery is another unanswered question. More experiments are needed to advance our understanding of the influence of non-invasive therapies within the meningeal interface.

## Synaptic signaling targets

Pathological signaling cascades of neurotrauma ultimately result in synaptic dysfunction. Understanding the mechanisms of synaptic dysfunction in TBI is crucial for developing therapeutic interventions that can mitigate these effects, protect neural tissues, and promote recovery. Ion channel dynamics is a target for improving synaptic dysfunction. The voltage-gated sodium channel Na_v_1.3 is upregulated following TBI (37, 38). Increased expression of the voltage-gated sodium channel leads to irregular neuronal excitability. Abnormalities in voltage-gated sodium, potassium and calcium channels have been associated with epilepsy (198, 199). In particular, Sodium channels Nav1.1, Nav1.2 and Nav1.3 play a key role in epileptic pathology (199). Indeed, this may be an avenue to reduce the risk of developing TBI-related epilepsy as well as other cognitive deficits. Modulating injury induced synaptic dysfunction via the regulation of ionic channel dynamics, such as Na_v1.3_ may be an effective strategy to stabilize the synapse and prevent abnormal generation of action potentials which lead to seizures as well as other cognitive deficits.

TBI disrupts calcium homeostasis and therefore calcium signaling is a key molecular target of non-invasive therapies. The initial injury induced influx of Ca^2+^ into the cell results in apoptosis. The cellular death, coupled with neural circuits dysregulation can lead to the excessive release of glutamate following neurotrauma (45, 46). This increase in glutamate then causes a further influx of Ca^2+^ through the NMDA receptor causing a secondary excitotoxic cascade (47). The increased Ca^2+^ ion within the cell is absorbed within the mitochondrial membrane (144). Which inhibits respiratory chain-linked electron transfer and energy transduction, impairing cellular function (144). Targeting of cytochrome c oxidase within the mitochondria has been shown to increase ATP production and rescue metabolic dysfunction (140, 141). Therefore, calcium homeostasis can be regulated through non-invasive therapy targeting mitochondrial function via cytochrome c oxidase activity.

Vascular endothelial cells are another site where calcium signaling can be targeted following TBI. Within the vascular endothelial cells, the mechanoreceptor Piezo1 senses mechanical force exerted on the cell membrane which leads to an influx of extracellular Ca^2+^ initiating an inflammatory signaling cascade (200). Apoptosis and injury-related changes in mechanical forces on the vascular endothelial cells can lead to the up-regulation of Piezo1 and a negative feedback loop of Ca^2+^ signaling (201). Changes within the microenvironment brought on injury, such as changes in blood flow and edema as well as a breakdown in tight-junction proteins can further affect the membrane mechanics therefore further activating Pizeo1 and accelerating inflammatory signaling (201). Piezo1 has therefore been suggested as an essential component for the transition of acute to chronic inflammation (200). Indeed, inhibiting Piezo1 has been shown to block the development of chronic inflammation. A future avenue of research could investigate the therapeutic benefit of targeting both cytochrome c oxidase within mitochondria as well as the Piezo1 channel within the vascular endothelial cells and astrocytic end feet help to regulate the Ca2+ signaling and subsequently preventing the secondary cascade and promoting recovery.

## Growth factor targets

Growth factors have been the focus of many treatments for neurodegenerative diseases, including TBI for several decades (202). The BDNF-TrkB signaling pathway is a key regulator of plasticity and neurogenesis and has therefore received a lot of attention as a potential therapeutic target (67, 68). Many of the neuromodulation techniques produced robust changes in BDNF expression or signaling. Indeed, in the case of ultrasound stimulation, the therapeutic effect was diminished when BDNF signaling was inhibited (137). Non-neuronal growth factors are also a target for non-invasive therapies. Factors such as VEGF are protective for endothelial cells during injury (137). In addition, the release of VEGF during the early post injury period promote the formation of new blood vessels, which in turn improves tissue oxygenation (73). Targeting of growth factors can initiate multiple neuroprotective mechanisms leading to a reduction in inflammatory cytokines and preserving cerebrovascular function. Treatment timing might determine which growth factor is most effective. Non-neuronal growth factors, such as VEGF, may be most effective during the early post injury period, providing protective properties and supporting the natural recovery processes, such as vasodilation and angiogenesis. Where BDNF-TrkB may be most effective at supporting the synapse recovery. The up regulation of growth factors is a key mechanism that leads to increased neurogenesis and enhanced cognition recovery. Therefore, the modulation of growth factors may be a key mechanism by which stimulation can induce lasting effects on plasticity and recovery after treatment ends.

## Translating preclinical findings

An important consideration is the degree that anesthesia affects the efficacy of non-invasive treatments. In many pre-clinical paradigm's rodents are anesthetized in order to administer treatment (105). Gersner et al., demonstrated that high frequency TMS had differing effects when administered to anesthetized rodents compared to awake animals (105). Combining head mounted miniaturized non-invasive stimulation with real time recording in awake behaving animals will help to delineate the effects of anesthesia and link neuronal function with therapeutic treatments. Furthermore, the field would be greatly advanced by establishing a common set of outcome measures used to monitor recovery, such as inflammation, blood-brain barrier integrity, synaptic function and behavior paradigms. This would create a common method by which different stimulation modalities and parameters could be compared.

## Summary

**Figure 6:**
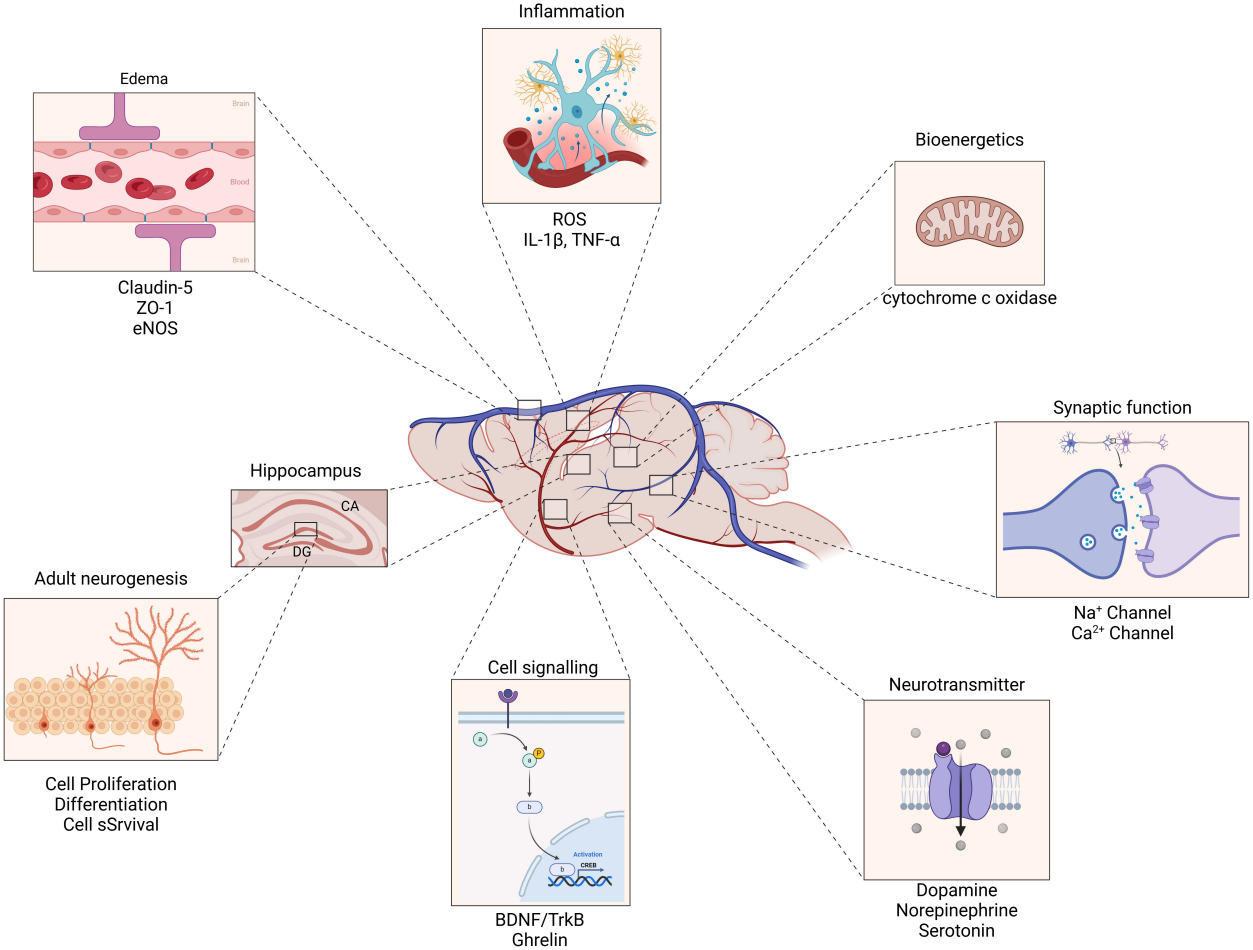
Mechanisms of action for non-invasive therapeutics in traumatic brain injury. Non-invasive therapies enhance the expression of Brain-Derived Neurotrophic Factor (BDNF) and its receptor, Tropomyosin receptor kinase B (TrkB), leading to improved synaptic plasticity and neuronal survival. This pathway plays a crucial role in the recovery and reorganization of neural networks following TBI. Therapeutics promote the proliferation and differentiation of neural progenitor cells in the hippocampus and other brain regions, contributing to the generation of new neurons. This process supports cognitive function and recovery of memory and learning abilities impaired by TBI. By modulating blood-brain barrier permeability, non-invasive interventions facilitate the clearance of excess fluid and inflammatory molecules from the brain parenchyma. This reduction in edema and inflammation mitigates secondary injury processes and supports overall neural health. The combined effects of these mechanisms underscore the potential of non-invasive therapeutic strategies to enhance recovery and improve outcomes in patients with traumatic brain injury.

TBI is a leading cause of death and disability with no proven interventions to improve patient outcome after brain injury. The development of novel therapies, such as non-invasive stimulation is a promising avenue for treatment, and different non-invasive treatments are being offered to TBI patients without a strong underlying premise. Preclinical research has the ability to give deeper insight into the potential mechanism of action and novel targets of non-invasive therapeutics for the treatment of TBI. Existing research already reveals that the therapeutic mechanisms of non-invasive therapies work through activating multiple neuroprotective mechanism which reduce edema and prevent blood-brain barrier breakdown and improve cerebrovascular dynamics (Figure 6). Furthermore, synaptic dysfunction is modulated through the regulation of ionic channel dynamics, driving LTP, transcriptional changes, upregulating growth factors and promoting adult hippocampal neurogenesis (Figure 6). Many mechanistic questions remain and more work is needed to identify key targets and their effective treatment time points in order to facilitate functional recovery. However, the combined effects of these mechanisms underscore the potential of non-invasive therapeutic strategies to enhance recovery and improve outcomes in patients with TBI.

## References

[1] Howlett JR Nelson LD Stein MB. Mental health consequences of traumatic brain injury. Biol Psychiatry. (2022) 91:413–20. 10.1016/j.biopsych.2021.09.024 34893317 PMC8849136

[2] Johnson WD Griswold DP. Traumatic brain injury: a global challenge. Lancet Neurol. (2017) 16:949–50. 10.1016/S1474-4422(17)30362-9 29122521

[3] Maas AI Menon DK Manley GT Abrams M Åkerlund C Andelic N. Traumatic brain injury: progress and challenges in prevention, clinical care, and research. Lancet Neurol. (2022) 21:1004–60. 10.1016/S1474-4422(22)00309-X 36183712 PMC10427240

[4] Jha RM Kochanek PM. Physiological trajectories after traumatic brain injury: markers or makers of disease? Lancet Neurol. (2024) 23:7–9. 10.1016/S1474-4422(23)00428-3 37977158 PMC11305088

[5] Paterno R Folweiler KA Cohen AS. Pathophysiology and treatment of memory dysfunction after traumatic brain injury. Curr Neurol Neurosci Rep. (2017) 17:52. 10.1007/s11910-017-0762-x 28500417 PMC5861722

[6] Hofer A-S Schwab ME. Enhancing rehabilitation and functional recovery after brain and spinal cord trauma with electrical neuromodulation. Curr Opin Neurol. (2019) 32:828–35. 10.1097/WCO.0000000000000750 31567546 PMC6855343

[7] Dhaliwal SK Meek BP Modirrousta MM. Non-invasive brain stimulation for the treatment of symptoms following traumatic brain injury. Front Psychiatry. (2015) 6:119. 10.3389/fpsyt.2015.00119 26379560 PMC4549551

[8] Popa LL Chira D Strilciuc Ş Mureşanu DF. Non-invasive systems application in traumatic brain injury rehabilitation. Brain Sci. (2023) 13:1594. 10.3390/brainsci13111594 38002552 PMC10670234

[9] Rudroff T Workman CD. Transcranial direct current stimulation as a treatment tool for mild traumatic brain injury. Brain Sci. (2021) 11:806. 10.3390/brainsci11060806 34207004 PMC8235194

[10] Galimberti A Tik M Pellegrino G Schuler A-L. Effectiveness of rTMS and tDCS treatment for chronic TBI symptoms: a systematic review and meta-analysis. Prog Neuropsychopharmacol Biol Psychiatry. (2024) 128:110863. 10.1016/j.pnpbp.2023.110863 37709126

[11] Campbell BCV Khatri P. Stroke. Lancet. (2020) 396:129–42. 10.1016/S0140-6736(20)31179-X 32653056

[12] Vidale S Consoli A Arnaboldi M Consoli D. Postischemic inflammation in acute stroke. J Clin Neurol. (2017) 13:1–9. 10.3988/jcn.2017.13.1.1 28079313 PMC5242162

[13] Maas AIR Menon DK Adelson PD Andelic N Bell MJ Belli A. Traumatic brain injury: integrated approaches to improve prevention, clinical care, and research. Lancet Neurol. (2017) 16:987–1048. 10.1016/S1474-4422(17)30371-X 29122524

[14] Langhorne P Bernhardt J Kwakkel G. Stroke rehabilitation. Lancet. (2011) 377:1693–702. 10.1016/S0140-6736(11)60325-5 21571152

[15] Miller KK Porter RE DeBaun-Sprague E Van Puymbroeck M Schmid AA. Exercise after stroke: patient adherence and beliefs after discharge from rehabilitation. Top Stroke Rehabil. (2017) 24:142–8. 10.1080/10749357.2016.1200292 27334684

[16] Bramlett HM Dietrich WD. Pathophysiology of cerebral ischemia and brain trauma: similarities and differences. J Cereb Blood Flow Metab. (2004) 24:133–50. 10.1097/01.WCB.0000111614.19196.04 14747740

[17] Werner C Engelhard K. Pathophysiology of traumatic brain injury. Br J Anaesth. (2007) 99:4–9. 10.1093/bja/aem131 17573392

[18] Khatri N Sumadhura B Kumar S Kaundal RK Sharma S Datusalia AK. The Complexity of secondary cascade consequent to traumatic brain injury: pathobiology and potential treatments. Curr Neuropharmacol. (2021) 19:1984–2011. 10.2174/1570159X19666210215123914 33588734 PMC9185786

[19] Burke JF Stulc JL Skolarus LE Sears ED Zahuranec DB Morgenstern LB. Traumatic brain injury may be an independent risk factor for stroke. Neurology. (2013) 81:33–9. 10.1212/WNL.0b013e318297eecf 23803315 PMC3770205

[20] Qu D Li W Zhang S Li R Wang H Chen B. Traumatic brain injury is associated with both hemorrhagic stroke and ischemic stroke: a systematic review and meta-analysis. Front Neurosci. (2022) 16:814684. 10.3389/fnins.2022.814684 35221904 PMC8867812

[21] Collaborators GBD Feigin VL Stark BA Johnson CO Roth GA Bisignano C. Global, regional, and national burden of stroke and its risk factors, 1990–2019: a systematic analysis for the Global Burden of Disease Study 2019. Lancet Neurol. (2021) 20:795–820. 10.1016/S1474-4422(21)00252-0 34487721 PMC8443449

[22] Kekic M Boysen E Campbell IC Schmidt U. A systematic review of the clinical efficacy of transcranial direct current stimulation (tDCS) in psychiatric disorders. J Psychiatr Res. (2016) 74:70–86. 10.1016/j.jpsychires.2015.12.018 26765514

[23] Bennabi D Pedron S Haffen E Monnin J Peterschmitt Y Van Waes V. Transcranial direct current stimulation for memory enhancement: from clinical research to animal models. Front Syst Neurosci. (2014) 8:159. 10.3389/fnsys.2014.00159 25237299 PMC4154388

[24] Brasil-Neto JP. Learning, memory, and transcranial direct current stimulation. Front Psychiatry. (2012) 3:80. 10.3389/fpsyt.2012.00080 22969734 PMC3432476

[25] de Vries MH Barth ACR Maiworm S Knecht S Zwitserlood P Flöel A. Electrical stimulation of Broca's area enhances implicit learning of an artificial grammar. J Cogn Neurosci. (2010) 22:2427–36. 10.1162/jocn.2009.21385 19925194

[26] Bolognini N Fregni F Casati C Olgiati E Vallar G. Brain polarization of parietal cortex augments training-induced improvement of visual exploratory and attentional skills. Brain Res. (2010) 1349:76–89. 10.1016/j.brainres.2010.06.053 20599813

[27] Galea JM Celnik P. Brain polarization enhances the formation and retention of motor memories. J Neurophysiol. (2009) 102:294–301. 10.1152/jn.00184.2009 19386757 PMC2712265

[28] Kincses TZ Antal A Nitsche MA Bártfai O Paulus W. Facilitation of probabilistic classification learning by transcranial direct current stimulation of the prefrontal cortex in the human. Neuropsychologia. (2004) 42:113–7. 10.1016/S0028-3932(03)00124-6 14615081

[29] Nitsche MA Fricke K Henschke U Schlitterlau A Liebetanz D Lang N. Pharmacological modulation of cortical excitability shifts induced by transcranial direct current stimulation in humans. J Physiol. (2003) 553:293–301. 10.1113/jphysiol.2003.049916 12949224 PMC2343495

[30] Pelletier SJ Cicchetti F. Cellular and molecular mechanisms of action of transcranial direct current stimulation: evidence from in vitro and in vivo models. Int J Neuropsychopharmacol. (2014) 18:pyu047. 10.1093/ijnp/pyu047 25522391 PMC4368894

[31] Vasu SO Kaphzan H. The role of sodium channels in direct current stimulation—axonal perspective. Cell Rep. (2021) 37:109832. 10.1016/j.celrep.2021.109832 34644580

[32] Monai H Ohkura M Tanaka M Oe Y Konno A Hirai H. Calcium imaging reveals glial involvement in transcranial direct current stimulation-induced plasticity in mouse brain. Nat Commun. (2016) 7:11100. 10.1038/ncomms11100 27000523 PMC4804173

[33] Liebetanz D Nitsche MA Tergau F Paulus W. Pharmacological approach to the mechanisms of transcranial DC-stimulation-induced after-effects of human motor cortex excitability. Brain. (2002) 125:2238–47. 10.1093/brain/awf238 12244081

[34] Mosayebi-Samani M Melo L Agboada D Nitsche MA Kuo M-F. Ca2+ channel dynamics explain the nonlinear neuroplasticity induction by cathodal transcranial direct current stimulation over the primary motor cortex. Eur Neuropsychopharmacol. (2020) 38:63–72. 10.1016/j.euroneuro.2020.07.011 32768154

[35] Sudbrack-Oliveira P Barbosa MZ Thome-Souza S Razza LB Gallucci-Neto J da Costa Lane Valiengo L. Transcranial direct current stimulation (tDCS) in the management of epilepsy: a systematic review. Seizure. (2021) 86:85–95. 10.1016/j.seizure.2021.01.020 33582584

[36] Lowenstein DH. Epilepsy after head injury: an overview. Epilepsia. (2009) 50:4–9. 10.1111/j.1528-1167.2008.02004.x 19187288

[37] Huang X-J Mao Q Lin Y Feng J Jiang J. Expression of voltage-gated sodium channel Nav1.3 is associated with severity of traumatic brain injury in adult rats. J Neurotrauma. (2013) 30:39–46. 10.1089/neu.2012.2508 22928478 PMC3530945

[38] Huang X-J Li W-P Lin Y Feng J-F Jia F Mao Q. Blockage of the upregulation of voltage-gated sodium channel nav1.3 improves outcomes after experimental traumatic brain injury. J Neurotrauma. (2014) 31:346–357. 10.1089/neu.2013.2899 24313291 PMC3922240

[39] Hu H-J Song M. Disrupted ionic homeostasis in ischemic stroke and new therapeutic targets. J Stroke Cerebrovasc Dis. (2017) 26:2706–19. 10.1016/j.jstrokecerebrovasdis.2017.09.011 29054733

[40] Chamorro Á Dirnagl U Urra X Planas AM. Neuroprotection in acute stroke: targeting excitotoxicity, oxidative and nitrosative stress, and inflammation. Lancet Neurol. (2016) 15:869–81. 10.1016/S1474-4422(16)00114-9 27180033

[41] Stagg CJ Best JG Stephenson MC O'Shea J Wylezinska M Kincses ZT. Polarity-sensitive modulation of cortical neurotransmitters by transcranial stimulation. J Neurosci. (2009) 29:5202–6. 10.1523/JNEUROSCI.4432-08.2009 19386916 PMC6665468

[42] Dirnagl U Iadecola C Moskowitz MA. Pathobiology of ischaemic stroke: an integrated view. Trends Neurosci. (1999) 22:391–7. 10.1016/S0166-2236(99)01401-0 10441299

[43] Peruzzotti-Jametti L Cambiaghi M Bacigaluppi M Gallizioli M Gaude E Mari S. Safety and efficacy of transcranial direct current stimulation in acute experimental ischemic stroke. Stroke. (2013) 44:3166–74. 10.1161/STROKEAHA.113.001687 23982710

[44] Chen Y-H Huang EY-K Kuo T-T Miller J Chiang Y-H Hoffer BJ. Impact of traumatic brain injury on dopaminergic transmission. Cell Transplant. (2017) 26:1156–68. 10.1177/0963689717714105 28933212 PMC5657731

[45] Dorsett CR McGuire JL DePasquale EAK Gardner AE Floyd CL McCullumsmith RE. Glutamate neurotransmission in rodent models of traumatic brain injury. J Neurotrauma. (2017) 34:263–72. 10.1089/neu.2015.4373 27256113 PMC5220558

[46] Guerriero RM Giza CC Rotenberg A. Glutamate and GABA imbalance following traumatic brain injury. Curr Neurol Neurosci Rep. (2015) 15:27. 10.1007/s11910-015-0545-1 25796572 PMC4640931

[47] Hoffe B Holahan MR. Hyperacute excitotoxic mechanisms and synaptic dysfunction involved in traumatic brain injury. Front Mol Neurosci. (2022) 15:831825. 10.3389/fnmol.2022.831825 35283730 PMC8907921

[48] Ikonomidou C Turski L. Why did NMDA receptor antagonists fail clinical trials for stroke and traumatic brain injury? Lancet Neurol. (2002) 1:383–6. 10.1016/S1474-4422(02)00164-3 12849400

[49] Barbati SA Podda MV Grassi C. Tuning brain networks: the emerging role of transcranial direct current stimulation on structural plasticity. Front Cell Neurosci. (2022) 16:945777. 10.3389/fncel.2022.945777 35936497 PMC9351051

[50] Almeida-Suhett CP Prager EM Pidoplichko V Figueiredo TH Marini AM Li Z. Reduced GABAergic inhibition in the basolateral amygdala and the development of anxiety-like behaviors after mild traumatic brain injury. PLoS ONE. (2014) 9:e102627. 10.1371/journal.pone.0102627 25047645 PMC4105413

[51] Delic V Beck KD Pang KCH Citron BA. Biological links between traumatic brain injury and Parkinson's disease. Acta Neuropathol Commun. (2020) 8:45. 10.1186/s40478-020-00924-7 32264976 PMC7137235

[52] Acosta SA Tajiri N de la Pena I Bastawrous M Sanberg PR Kaneko Y. Alpha-synuclein as a pathological link between chronic traumatic brain injury and Parkinson's disease. J Cell Physiol. (2015) 230:1024–1032. 10.1002/jcp.24830 25251017 PMC4328145

[53] Impellizzeri D Campolo M Bruschetta G Crupi R Cordaro M Paterniti I. Traumatic brain injury leads to development of parkinson's disease related pathology in mice. Front Neurosci. (2016) 10:458. 10.3389/fnins.2016.00458 27790086 PMC5061819

[54] Fukai M Bunai T Hirosawa T Kikuchi M Ito S Minabe Y. Endogenous dopamine release under transcranial direct-current stimulation governs enhanced attention: a study with positron emission tomography. Transl Psychiatry. (2019) 9:115. 10.1038/s41398-019-0443-4 30877269 PMC6420561

[55] Kornhuber J Weller M. Psychotogenicity and N-methyl-D-aspartate receptor antagonism: implications for neuroprotective pharmacotherapy. Biol Psychiatry. (1997) 41:135–44. 10.1016/S0006-3223(96)00047-9 9018383

[56] Loggini A Tangonan R El Ammar F Mansour A Goldenberg FD Kramer CL. The role of amantadine in cognitive recovery early after traumatic brain injury: a systematic review. Clin Neurol Neurosurg. (2020) 194:105815. 10.1016/j.clineuro.2020.105815 32244036

[57] Snyder JS Drew MR. Functional neurogenesis over the years. Behav Brain Res. (2020) 382:112470. 10.1016/j.bbr.2020.112470 31917241 PMC7769695

[58] Toda T Parylak SL Linker SB Gage FH. The role of adult hippocampal neurogenesis in brain health and disease. Mol Psychiatry. (2019) 24:67–87. 10.1038/s41380-018-0036-2 29679070 PMC6195869

[59] Redell JB Maynard ME Underwood EL Vita SM Dash PK Kobori N. Traumatic brain injury and hippocampal neurogenesis: functional implications. Exp Neurol. (2020) 331:113372. 10.1016/j.expneurol.2020.113372 32504636 PMC7803458

[60] Dash PK Mach SA Moore AN. Enhanced neurogenesis in the rodent hippocampus following traumatic brain injury. J Neurosci Res. (2001) 63:313–9. 10.1002/1097-4547(20010215)63:4<313::AID-JNR1025>3.0.CO;2-4 PMID: 11170181 11170181

[61] Neuberger EJ Swietek B Corrubia L Prasanna A Santhakumar V. Enhanced dentate neurogenesis after brain injury undermines long-term neurogenic potential and promotes seizure susceptibility. Stem Cell Rep. (2017) 9:972–84. 10.1016/j.stemcr.2017.07.015 28826852 PMC5599224

[62] Ratliff WA Delic V Pick CG Citron BA. Dendritic arbor complexity and spine density changes after repetitive mild traumatic brain injury and neuroprotective treatments. Brain Res. (2020) 1746:147019. 10.1016/j.brainres.2020.147019 32681835 PMC8994668

[63] Pikhovych A Stolberg NP Jessica Flitsch L Walter HL Graf R Fink GR. Transcranial direct current stimulation modulates neurogenesis and microglia activation in the mouse brain. Stem Cells Int. (2016) 2016:2715196. 10.1155/2016/2715196 27403166 PMC4925996

[64] Yu T-H Wu Y-J Chien M-E Hsu K-S. Multisession anodal transcranial direct current stimulation enhances adult hippocampal neurogenesis and context discrimination in mice. J Neurosci. (2023) 43:635–46. 10.1523/JNEUROSCI.1476-22.2022 36639896 PMC9888513

[65] Braun R Klein R Walter HL Ohren M Freudenmacher L Getachew K. Transcranial direct current stimulation accelerates recovery of function, induces neurogenesis and recruits oligodendrocyte precursors in a rat model of stroke. Exp Neurol. (2016) 279:127–36. 10.1016/j.expneurol.2016.02.018 26923911

[66] Sukenik N Vinogradov O Weinreb E Segal M Levina A Moses E. Neuronal circuits overcome imbalance in excitation and inhibition by adjusting connection numbers. Proc Nat Acad Sci. (2021) 118:e2018459118. 10.1073/pnas.2018459118 33723048 PMC8000583

[67] Cocco S Podda MV Grassi C. Role of BDNF signaling in memory enhancement induced by transcranial direct current stimulation. Front Neurosci. (2018) 12:427. 10.3389/fnins.2018.00427 29997473 PMC6028595

[68] Longo V Barbati SA Re A Paciello F Bolla M Rinaudo M. Transcranial direct current stimulation enhances neuroplasticity and accelerates motor recovery in a stroke mouse model. Stroke. (2022) 53:1746–58. 10.1161/STROKEAHA.121.034200 35291824

[69] Yoon KJ Lee Y-T Chae SW Park CR Kim DY. Effects of anodal transcranial direct current stimulation (tDCS) on behavioral and spatial memory during the early stage of traumatic brain injury in the rats. J Neurol Sci. (2016) 362:314–20. 10.1016/j.jns.2016.02.005 26944170

[70] Fritsch B Reis J Martinowich K Schambra HM Ji Y Cohen LG. Direct current stimulation promotes BDNF-dependent synaptic plasticity: potential implications for motor learning. Neuron. (2010) 66:198–204. 10.1016/j.neuron.2010.03.035 20434997 PMC2864780

[71] Ahn SM Jung DH Lee HJ Pak ME Jung YJ Shin Y-I. Contralesional application of transcranial direct current stimulation on functional improvement in ischemic stroke mice. Stroke. (2020) 51:2208–18. 10.1161/STROKEAHA.120.029221 32521221

[72] Aaron RK Boyan BD Ciombor DM Schwartz Z Simon BJ. Stimulation of growth factor synthesis by electric and electromagnetic fields. Clin Orthop Relat Res. (2004) 419:30–7. 10.1097/00003086-200402000-00006 15021128

[73] Bragina OA Lara DA Nemoto EM Shuttleworth CW Semyachkina-Glushkovskaya OV Bragin DE. Increases in microvascular perfusion and tissue oxygenation via vasodilatation after anodal transcranial direct current stimulation in the healthy and traumatized mouse brain. Adv Exp Med Biol. (2018) 1072:27–31. 10.1007/978-3-319-91287-5_5 30178319 PMC6294145

[74] Bragina OA Semyachkina-Glushkovskaya OV Nemoto EM Bragin DE. Anodal transcranial direct current stimulation improves impaired cerebrovascular reactivity in traumatized mouse brain. Adv Exp Med Biol. (2020) 1232:47–53. 10.1007/978-3-030-34461-0_7 31893393 PMC7307636

[75] Akcay G Demirdogen F Gul T Yilmaz A Kotan D Karakoc E. Effects of transcranial direct current stimulation on motor and cognitive dysfunction in an experimental traumatic brain injury model. Turk Neurosurg. (2024) 34:343–50. 10.5137/1019-5149.JTN.45526-23.4 38497188

[76] Reato D Rahman A Bikson M Parra LC. Effects of weak transcranial alternating current stimulation on brain activity—a review of known mechanisms from animal studies. Front Hum Neurosci. (2013) 7. 10.3389/fnhum.2013.00687 24167483 PMC3805939

[77] Ward LM. Synchronous neural oscillations and cognitive processes. Trends Cogn Sci. (2003) 7:553–9. 10.1016/j.tics.2003.10.012 14643372

[78] Uhlhaas PJ Singer W. Neural synchrony in brain disorders: relevance for cognitive dysfunctions and pathophysiology. Neuron. (2006) 52:155–68. 10.1016/j.neuron.2006.09.020 17015233

[79] Koch PF Cottone C Adam CD Ulyanova AV Russo RJ Weber MT. Traumatic brain injury preserves firing rates but disrupts laminar oscillatory coupling and neuronal entrainment in hippocampal CA1. eNeuro. (2020) 7:ENEURO.0495-19.2020. 10.1523/ENEURO.0495-19.2020 32737188 PMC7477953

[80] Yavari F Jamil A Mosayebi Samani M Vidor LP Nitsche MA. Basic and functional effects of transcranial Electrical Stimulation (tES)—an introduction. Neurosci Biobehav Rev. (2018) 85:81–92. 10.1016/j.neubiorev.2017.06.015 28688701

[81] Antal A Paulus W. Transcranial alternating current stimulation (tACS). Front Hum Neurosci. (2013) 7:317. 10.3389/fnhum.2013.00317 23825454 PMC3695369

[82] Kim J Kim H Jeong H Roh D Kim DH. tACS as a promising therapeutic option for improving cognitive function in mild cognitive impairment: a direct comparison between tACS and tDCS. J Psychiatr Res. (2021) 141:248–56. 10.1016/j.jpsychires.2021.07.012 34256276

[83] Hallett M. Transcranial magnetic stimulation and the human brain. Nature. (2000) 406:147–50. 10.1038/35018000 10910346

[84] Nardone R Sebastianelli L Versace V Brigo F Golaszewski S Manganotti P. Repetitive transcranial magnetic stimulation in traumatic brain injury: evidence from animal and human studies. Brain Res Bull. (2020) 159:44–52. 10.1016/j.brainresbull.2020.03.016 32251693

[85] Pape TL-B Rosenow J Lewis G. Transcranial magnetic stimulation: a possible treatment for TBI. J Head Trauma Rehabil. (2006) 21:437–51. 10.1097/00001199-200609000-00063 16983227

[86] Chervyakov AV Chernyavsky AY Sinitsyn DO Piradov MA. Possible mechanisms underlying the therapeutic effects of transcranial magnetic stimulation. Front Hum Neurosci. (2015) 9:303. 10.3389/fnhum.2015.00303 26136672 PMC4468834

[87] Hallett M. Transcranial magnetic stimulation: a primer. Neuron. (2007) 55:187–99. 10.1016/j.neuron.2007.06.026 17640522

[88] Houdayer E Degardin A Cassim F Bocquillon P Derambure P Devanne H. The effects of low- and high-frequency repetitive TMS on the input/output properties of the human corticospinal pathway. Exp Brain Res. (2008) 187:207–17. 10.1007/s00221-008-1294-z 18259738

[89] Huang Y-Z Edwards MJ Rounis E Bhatia KP Rothwell JC. Theta burst stimulation of the human motor cortex. Neuron. (2005) 45:201–6. 10.1016/j.neuron.2004.12.033 15664172

[90] Lu H Kobilo T Robertson C Tong S Celnik P Pelled G. Transcranial magnetic stimulation facilitates neurorehabilitation after pediatric traumatic brain injury. Sci Rep. (2015) 5:14769. 10.1038/srep14769 26440604 PMC4594036

[91] Hsu W-Y Cheng C-H Liao K-K Lee I-H Lin Y-Y. Effects of repetitive transcranial magnetic stimulation on motor functions in patients with stroke: a meta-analysis. Stroke. (2012) 43:1849–57. 10.1161/STROKEAHA.111.649756 22713491

[92] Chiang C-F Lin M-T Hsiao M-Y Yeh Y-C Liang Y-C Wang T-G. Comparative efficacy of noninvasive neurostimulation therapies for acute and subacute poststroke dysphagia: a systematic review and network meta-analysis. Arch Phys Med Rehabil. (2019) 100:739–50. 10.1016/j.apmr.2018.09.117 30352222

[93] Yin M Liu Y Zhang L Zheng H Peng L Ai Y. Effects of rTMS treatment on cognitive impairment and resting-state brain activity in stroke patients: a randomized clinical trial. Front Neural Circuits. (2020) 14:563777. 10.3389/fncir.2020.563777 33117131 PMC7561423

[94] Jorge RE Robinson RG Tateno A Narushima K Acion L Moser D. Repetitive transcranial magnetic stimulation as treatment of poststroke depression: a preliminary study. Biol Psychiatry. (2004) 55:398–405. 10.1016/j.biopsych.2003.08.017 14960293

[95] Lai TW Zhang S Wang YT. Excitotoxicity and stroke: identifying novel targets for neuroprotection. Prog Neurobiol. (2014) 115:157–88. 10.1016/j.pneurobio.2013.11.006 24361499

[96] Ikeda T Kobayashi S Morimoto C. Effects of repetitive transcranial magnetic stimulation on ER stress-related genes and glutamate, γ-aminobutyric acid and glycine transporter genes in mouse brain. Biochem Biophys Rep. (2019) 17:10–6. 10.1016/j.bbrep.2018.10.015 30456316 PMC6234257

[97] Zangen A Hyodo K. Transcranial magnetic stimulation induces increases in extracellular levels of dopamine and glutamate in the nucleus accumbens. Neuroreport. (2002) 13:2401–5. 10.1097/00001756-200212200-00005 12499837

[98] McNerney MW Heath A Narayanan SK Yesavage J. Repetitive transcranial magnetic stimulation improves brain-derived neurotrophic factor and cholinergic signaling in the 3xTgAD mouse model of Alzheimer's disease. J Alzheimers Dis. (2022) 86:499–507. 10.3233/JAD-215361 35068462 PMC9028616

[99] Lefaucheur J-P Drouot X Von Raison F Ménard-Lefaucheur I Cesaro P Nguyen J-P. Improvement of motor performance and modulation of cortical excitability by repetitive transcranial magnetic stimulation of the motor cortex in Parkinson's disease. Clin Neurophysiol. (2004) 115:2530–41. 10.1016/j.clinph.2004.05.025 15465443

[100] Atkins CM. Decoding hippocampal signaling deficits after traumatic brain injury. Transl Stroke Res. (2011) 2:546–55. 10.1007/s12975-011-0123-z 23227133 PMC3514866

[101] Schwarzbach E Bonislawski DP Xiong G Cohen AS. Mechanisms underlying the inability to induce area CA1 LTP in the mouse after traumatic brain injury. Hippocampus. (2006) 16:541–50. 10.1002/hipo.20183 16634077 PMC3951737

[102] Chang WH Bang OY Shin Y-I Lee A Pascual-Leone A Kim Y-H. BDNF polymorphism and differential rTMS effects on motor recovery of stroke patients. Brain Stimul. (2014) 7:553–8. 10.1016/j.brs.2014.03.008 24767962

[103] Wang H-Y Crupi D Liu J Stucky A Cruciata G Di Rocco A. Repetitive transcranial magnetic stimulation enhances BDNF-TrkB signaling in both brain and lymphocyte. J Neurosci. (2011) 31:11044–54. 10.1523/JNEUROSCI.2125-11.2011 21795553 PMC3161730

[104] Marshall J Szmydynger-Chodobska J Rioult-Pedotti MS Lau K Chin AT Kotla SKR. TrkB-enhancer facilitates functional recovery after traumatic brain injury. Sci Rep. (2017) 7:10995. 10.1038/s41598-017-11316-8 28887487 PMC5591207

[105] Gersner R Kravetz E Feil J Pell G Zangen A. Long-term effects of repetitive transcranial magnetic stimulation on markers for neuroplasticity: differential outcomes in anesthetized and awake animals. J Neurosci. (2011) 31:7521–6. 10.1523/JNEUROSCI.6751-10.2011 21593336 PMC6622610

[106] Vahabzadeh-Hagh AM Muller PA Gersner R Zangen A Rotenberg A. Translational neuromodulation: approximating human transcranial magnetic stimulation protocols in rats. Neuromodulation. (2012) 15:296–305. 10.1111/j.1525-1403.2012.00482.x 22780329 PMC5764706

[107] Choung JS Bhattacharjee S Son JP Kim JM Cho DS Cho CS. Development and application of rTMS device to murine model. Sci Rep. (2023) 13:5490. 10.1038/s41598-023-32646-w 37016000 PMC10073209

[108] Jiang W Isenhart R Liu CY Song D. A C-shaped miniaturized coil for transcranial magnetic stimulation in rodents. J Neural Eng. (2023) 20:acc097. 10.1088/1741-2552/acc097 36863013 PMC10037933

[109] Nieminen JO Pospelov AS Koponen LM Yrjölä P Shulga A Khirug S. Transcranial magnetic stimulation set-up for small animals. Front Neurosci. (2022) 16:935268. 10.3389/fnins.2022.935268 36440290 PMC9685557

[110] Sekar S Zhang Y Miranzadeh Mahabadi H Parvizi A Taghibiglou C. Low-field magnetic stimulation restores cognitive and motor functions in the mouse model of repeated traumatic brain injury: role of cellular prion protein. J Neurotrauma. (2019) 36:3103–14. 10.1089/neu.2018.5918 31020907

[111] Sasso V Bisicchia E Latini L Ghiglieri V Cacace F Carola V. Repetitive transcranial magnetic stimulation reduces remote apoptotic cell death and inflammation after focal brain injury. J Neuroinflammation. (2016) 13:150. 10.1186/s12974-016-0616-5 27301743 PMC4908713

[112] Toledo RS Stein DJ Stefani Sanches PR de Souza A da Silva LS Medeiros HR. Repetitive transcranial magnetic stimulation (rTMS) reverses the long-term memory impairment and the decrease of hippocampal interleukin-10 levels, both induced by neuropathic pain in rats. Neuroscience. (2021) 472:51–9. 10.1016/j.neuroscience.2021.07.030 34358630

[113] Zong X Li Y Liu C Qi W Han D Tucker L. Theta-burst transcranial magnetic stimulation promotes stroke recovery by vascular protection and neovascularization. Theranostics. (2020) 10:12090–110. 10.7150/thno.51573 33204331 PMC7667689

[114] Hu Y-Y Yang G Liang X-S Ding X-S Xu D-E Li Z. Transcranial low-intensity ultrasound stimulation for treating central nervous system disorders: a promising therapeutic application. Front Neurol. (2023) 14:1117188. 10.3389/fneur.2023.1117188 36970512 PMC10030814

[115] Ranade SS Syeda R Patapoutian A. Mechanically activated ion channels. Neuron. (2015) 87:1162–79. 10.1016/j.neuron.2015.08.032 26402601 PMC4582600

[116] Tyler WJ. The mechanobiology of brain function. Nat Rev Neurosci. (2012) 13:867–78. 10.1038/nrn3383 23165263

[117] Qiu Z Guo J Kala S Zhu J Xian Q Qiu W. The mechanosensitive ion channel piezo1 significantly mediates *in vitro* ultrasonic stimulation of neurons. iScience. (2019) 21:448–57. 10.1016/j.isci.2019.10.037 31707258 PMC6849147

[118] Zhu J Xian Q Hou X Wong KF Zhu T Chen Z. The mechanosensitive ion channel Piezo1 contributes to ultrasound neuromodulation. Proc Natl Acad Sci U S A. (2023) 120:e2300291120. 10.1073/pnas.2300291120 37098060 PMC10161134

[119] Coste B Mathur J Schmidt M Earley TJ Ranade S Petrus MJ. Piezo1 and Piezo2 are essential components of distinct mechanically activated cation channels. Science. (2010) 330:55–60. 10.1126/science.1193270 20813920 PMC3062430

[120] Yang S Miao X Arnold S Li B Ly AT Wang H. Membrane curvature governs the distribution of Piezo1 in live cells. Nat Commun. (2022) 13:7467. 10.1038/s41467-022-35034-6 36463216 PMC9719557

[121] Lim XR Abd-Alhaseeb MM Ippolito M Koide M Senatore AJ Plante C. Endothelial Piezo1 channel mediates mechano-feedback control of brain blood flow. Nat Commun. (2024) 15:8686. 10.1038/s41467-024-52969-0 39375369 PMC11458797

[122] Choi D Park E Choi J Lu R Yu JS Kim C. Piezo1 regulates meningeal lymphatic vessel drainage and alleviates excessive CSF accumulation. Nat Neurosci. (2024) 27:913–26. 10.1038/s41593-024-01604-8 38528202 PMC11088999

[123] Loane DJ Pocivavsek A Moussa CE-H Thompson R Matsuoka Y Faden AI. Amyloid precursor protein secretases as therapeutic targets for traumatic brain injury. Nat Med. (2009) 15:377–9. 10.1038/nm.1940 19287391 PMC2844765

[124] Drabik D Chodaczek G Kraszewski S. Effect of amyloid-β monomers on lipid membrane mechanical parameters-potential implications for mechanically driven neurodegeneration in Alzheimer's disease. Int J Mol Sci. (2020) 22. 10.3390/ijms22010018 33375009 PMC7792773

[125] Johnson VE Stewart W Smith DH. Traumatic brain injury and amyloid-β pathology: a link to Alzheimer's disease? Nat Rev Neurosci. (2010) 11:361–70. 10.1038/nrn2808 20216546 PMC3979339

[126] Maneshi MM Ziegler L Sachs F Hua SZ Gottlieb PA. Enantiomeric Aβ peptides inhibit the fluid shear stress response of PIEZO1. Sci Rep. (2018) 8:14267. 10.1038/s41598-018-32572-2 30250223 PMC6155315

[127] Chirila AM Rankin G Tseng S-Y Emanuel AJ Chavez-Martinez CL Zhang D. Mechanoreceptor signal convergence and transformation in the dorsal horn flexibly shape a diversity of outputs to the brain. Cell. (2022) 185:4541–9.e23. 10.1016/j.cell.2022.10.012 36334588 PMC9691598

[128] Lehnert BP Santiago C Huey EL Emanuel AJ Renauld S Africawala N. Mechanoreceptor synapses in the brainstem shape the central representation of touch. Cell. (2021) 184:5608–21.e18. 10.1016/j.cell.2021.09.023 34637701 PMC8556359

[129] Main BS Villapol S Sloley SS Barton DJ Parsadanian M Agbaegbu C. Apolipoprotein E4 impairs spontaneous blood brain barrier repair following traumatic brain injury. Mol Neurodegener. (2018) 13:17. 10.1186/s13024-018-0249-5 29618365 PMC5885297

[130] Chodobski A Zink BJ Szmydynger-Chodobska J. Blood-brain barrier pathophysiology in traumatic brain injury. Transl Stroke Res. (2011) 2:492–516. 10.1007/s12975-011-0125-x 22299022 PMC3268209

[131] Su W-S Tsai M-L Huang S-L Liu S-H Yang F-Y. Controllable permeability of blood-brain barrier and reduced brain injury through low-intensity pulsed ultrasound stimulation. Oncotarget. (2015) 6:42290–9. 10.18632/oncotarget.5978 26517350 PMC4747225

[132] Chen S-F Su W-S Wu C-H Lan T-H Yang F-Y. Transcranial ultrasound stimulation improves long-term functional outcomes and protects against brain damage in traumatic brain injury. Mol Neurobiol. (2018) 55:7079–89. 10.1007/s12035-018-0897-z 29383687

[133] Yoon SH Kwon SK Park SR Min B-H. Effect of ultrasound treatment on brain edema in a traumatic brain injury model with the weight drop method. Pediatr Neurosurg. (2012) 48:102–8. 10.1159/000343011 23154513

[134] Zheng T Du J Yuan Y Wu S Jin Y Wang Z. Neuroprotective effect of low-intensity transcranial ultrasound stimulation in moderate traumatic brain injury rats. Front Neurosci. (2020) 14:172. 10.3389/fnins.2020.00172 32218720 PMC7078644

[135] Huang L Kang J Chen G Ye W Meng X Du Q. Low-intensity focused ultrasound attenuates early traumatic brain injury by OX-A/NF-κB/NLRP3 signaling pathway. Aging. (2022) 14:7455–69. 10.18632/aging.204290 36126193 PMC9550253

[136] Mishima T Komano K Tabaru M Kofuji T Saito A Ugawa Y. Repetitive pulsed-wave ultrasound stimulation suppresses neural activity by modulating ambient GABA levels via effects on astrocytes. Front Cell Neurosci. (2024) 18:1361242. 10.3389/fncel.2024.1361242 38601023 PMC11004293

[137] Su W-S Wu C-H Chen S-F Yang F-Y. Transcranial ultrasound stimulation promotes brain-derived neurotrophic factor and reduces apoptosis in a mouse model of traumatic brain injury. Brain Stimul. (2017) 10:1032–41. 10.1016/j.brs.2017.09.003 28939348

[138] Liu S-H Lai Y-L Chen B-L Yang F-Y. Ultrasound enhances the expression of brain-derived neurotrophic factor in astrocyte through activation of TrkB-Akt and calcium-CaMK signaling pathways. Cereb Cortex. (2017) 27:3152–60. 10.1093/cercor/bhw169 27252349

[139] Nizamutdinov D Ezeudu C Wu E Huang JH Yi SS. Transcranial near-infrared light in treatment of neurodegenerative diseases. Front Pharmacol. (2022) 13:965788. 10.3389/fphar.2022.965788 36034819 PMC9400541

[140] Hennessy M Hamblin MR. Photobiomodulation and the brain: a new paradigm. J Opt. (2017) 19:013003. 10.1088/2040-8986/19/1/013003 28580093 PMC5448311

[141] Rojas JC Gonzalez-Lima F. Neurological and psychological applications of transcranial lasers and LEDs. Biochem Pharmacol. (2013) 86:447–57. 10.1016/j.bcp.2013.06.012 23806754

[142] Cassano P Petrie SR Hamblin MR Henderson TA Iosifescu DV. Review of transcranial photobiomodulation for major depressive disorder: targeting brain metabolism, inflammation, oxidative stress, and neurogenesis. Neurophotonics. (2016) 3:031404. 10.1117/1.NPh.3.3.031404 26989758 PMC4777909

[143] Mochizuki-Oda N Kataoka Y Cui Y Yamada H Heya M Awazu K. Effects of near-infra-red laser irradiation on adenosine triphosphate and adenosine diphosphate contents of rat brain tissue. Neurosci Lett. (2002) 323:207–10. 10.1016/S0304-3940(02)00159-3 11959421

[144] Xiong Y Gu Q Peterson PL Muizelaar JP Lee CP. Mitochondrial dysfunction and calcium perturbation induced by traumatic brain injury. J Neurotrauma. (1997) 14:23–34. 10.1089/neu.1997.14.23 9048308

[145] Hubbard WB Harwood CL Geisler JG Vekaria HJ Sullivan PG. Mitochondrial uncoupling prodrug improves tissue sparing, cognitive outcome, and mitochondrial bioenergetics after traumatic brain injury in male mice. J Neurosci Res. (2018) 96:1677–88. 10.1002/jnr.24271 30063076 PMC6129401

[146] Oron U Yaakobi T Oron A Mordechovitz D Shofti R Hayam G. Low-energy laser irradiation reduces formation of scar tissue after myocardial infarction in rats and dogs. Circulation. (2001) 103:296–301. 10.1161/01.CIR.103.2.296 11208692

[147] Killen MJ Giorgi-Coll S Helmy A Hutchinson PJ Carpenter KL. Metabolism and inflammation: implications for traumatic brain injury therapeutics. Expert Rev Neurother. (2019) 19:227–42. 10.1080/14737175.2019.1582332 30848963

[148] Förstermann U. Nitric oxide and oxidative stress in vascular disease. Pflugers Arch. (2010) 459:923–39. 10.1007/s00424-010-0808-2 20306272

[149] Abdul-Muneer PM Schuetz H Wang F Skotak M Jones J Gorantla S. Induction of oxidative and nitrosative damage leads to cerebrovascular inflammation in an animal model of mild traumatic brain injury induced by primary blast. Free Radic Biol Med. (2013) 60:282–91. 10.1016/j.freeradbiomed.2013.02.029 23466554 PMC4007171

[150] Zhang Q-G Laird MD Han D Nguyen K Scott E Dong Y. Critical role of NADPH oxidase in neuronal oxidative damage and microglia activation following traumatic brain injury. PLoS ONE. (2012) 7:e34504. 10.1371/journal.pone.0034504 22485176 PMC3317633

[151] Fesharaki-Zadeh A. Oxidative stress in traumatic brain injury. Int J Mol Sci. (2022) 23:13000. 10.3390/ijms232113000 36361792 PMC9657447

[152] Juan CA Pérez de la Lastra JM Plou FJ Pérez-Lebeña E. The chemistry of reactive oxygen species (ROS) revisited: outlining their role in biological macromolecules (DNA, lipids and proteins) and induced pathologies. Int J Mol Sci. (2021) 22:4642. 10.3390/ijms22094642 33924958 PMC8125527

[153] El Bakkouri Y Chidiac R Delisle C Corriveau J Cagnone G Gaonac'h-Lovejoy V. ZO-1 interacts with YB-1 in endothelial cells to regulate stress granule formation during angiogenesis. Nat Commun. (2024) 15:4405. 10.1038/s41467-024-48852-7 38782923 PMC11116412

[154] Jiao H Wang Z Liu Y Wang P Xue Y. Specific role of tight junction proteins claudin-5, occludin, and ZO-1 of the blood-brain barrier in a focal cerebral ischemic insult. J Mol Neurosci. (2011) 44:130–9. 10.1007/s12031-011-9496-4 21318404

[155] Huang N.agata K, Tedford CE, McCarthy T, Hamblin MR. Low-level laser therapy (LLLT) reduces oxidative stress in primary cortical neurons *in vitro*. J Biophotonics. (2013) 6:829–38. 10.1002/jbio.201200157 23281261 PMC3651776

[156] Förstermann U Sessa WC. Nitric oxide synthases: regulation and function. Eur Heart J. (2012) 33:829–37, 837a–d. 10.1093/eurheartj/ehr304 21890489 PMC3345541

[157] Garry PS Ezra M Rowland MJ Westbrook J Pattinson KTS. The role of the nitric oxide pathway in brain injury and its treatment–from bench to bedside. Exp Neurol. (2015) 263:235–43. 10.1016/j.expneurol.2014.10.017 25447937

[158] Kashiwagi S Morita A Yokomizo S Ogawa E Komai E Huang PL. Photobiomodulation and nitric oxide signaling. Nitric Oxide. (2023) 130:58–68. 10.1016/j.niox.2022.11.005 36462596 PMC9808891

[159] Thunshelle C Hamblin MR. Transcranial low-level laser (light) therapy for brain injury. Photomed Laser Surg. (2016) 34:587–98. 10.1089/pho.2015.4051 28001759 PMC5180077

[160] Xuan W Vatansever F Huang L Wu Q Xuan Y Dai T. Transcranial low-level laser therapy improves neurological performance in traumatic brain injury in mice: effect of treatment repetition regimen. PLoS ONE. (2013) 8:e53454. 10.1371/journal.pone.0053454 23308226 PMC3538543

[161] Xuan W Agrawal T Huang L Gupta GK Hamblin MR. Low-level laser therapy for traumatic brain injury in mice increases brain derived neurotrophic factor (BDNF) and synaptogenesis. J Biophotonics. (2015) 8:502–11. 10.1002/jbio.201400069 25196192 PMC5379854

[162] Ando T Xuan W Xu T Dai T Sharma SK Kharkwal GB. Comparison of therapeutic effects between pulsed and continuous wave 810-nm wavelength laser irradiation for traumatic brain injury in mice. PLoS ONE. (2011) 6:e26212. 10.1371/journal.pone.0026212 22028832 PMC3196530

[163] Oron A Oron U Streeter J de Taboada L Alexandrovich A Trembovler V. low-level laser therapy applied transcranially to mice following traumatic brain injury significantly reduces long-term neurological deficits. J Neurotrauma. (2007) 24:651–6. 10.1089/neu.2006.0198 17439348

[164] Wu Q Xuan W Ando T Xu T Huang L Huang Y-Y. Low-level laser therapy for closed-head traumatic brain injury in mice: effect of different wavelengths. Lasers Surg Med. (2012) 44:218–26. 10.1002/lsm.22003 22275301 PMC3397203

[165] Aburto MR Cryan JF. Gastrointestinal and brain barriers: unlocking gates of communication across the microbiota-gut-brain axis. Nat Rev Gastroenterol Hepatol. (2024) 21:222–47. 10.1038/s41575-023-00890-0 38355758

[166] Bonaz B Bazin T Pellissier S. The vagus nerve at the interface of the microbiota-gut-brain axis. Front Neurosci. (2018) 12:49. 10.3389/fnins.2018.00049 29467611 PMC5808284

[167] Neren D Johnson MD Legon W Bachour SP Ling G Divani AA. Vagus nerve stimulation and other neuromodulation methods for treatment of traumatic brain injury. Neurocrit Care. (2016) 24:308–19. 10.1007/s12028-015-0203-0 26399249

[168] Schachter SC Saper CB. Vagus nerve stimulation. Epilepsia. (1998) 39:677–86. 10.1111/j.1528-1157.1998.tb01151.x 9670894

[169] Andalib S Divani AA Ayata C Baig S Arsava EM Topcuoglu MA. Vagus nerve stimulation in ischemic stroke. Curr Neurol Neurosci Rep. (2023) 23:947–62. 10.1007/s11910-023-01323-w 38008851 PMC10841711

[170] Zhang H Li C-L Qu Y Yang Y-X Du J Zhao Y. Effects and neuroprotective mechanisms of vagus nerve stimulation on cognitive impairment with traumatic brain injury in animal studies: a systematic review and meta-analysis. Front Neurol. (2022) 13:963334. 10.3389/fneur.2022.963334 36237612 PMC9551312

[171] Tang Y Dong X Chen G Ye W Kang J Tang Y. Vagus nerve stimulation attenuates early traumatic brain injury by regulating the NF-κB/NLRP3 signaling pathway. Neurorehabil Neural Repair. (2020) 34:831–43. 10.1177/1545968320948065 32772884

[172] Clough RW Neese SL Sherill LK Tan AA Duke A Roosevelt RW. Cortical edema in moderate fluid percussion brain injury is attenuated by vagus nerve stimulation. Neuroscience. (2007) 147:286–93. 10.1016/j.neuroscience.2007.04.043 17543463

[173] Smith DC Tan AA Duke A Neese SL Clough RW Browning RA. Recovery of function after vagus nerve stimulation initiated 24 hours after fluid percussion brain injury. J Neurotrauma. (2006) 23:1549–60. 10.1089/neu.2006.23.1549 17020489

[174] Smith DC Modglin AA Roosevelt RW Neese SL Jensen RA Browning RA. Electrical stimulation of the vagus nerve enhances cognitive and motor recovery following moderate fluid percussion injury in the rat. J Neurotrauma. (2005) 22:1485–502. 10.1089/neu.2005.22.1485 16379585 PMC1769332

[175] Zhao Z-A Li P Ye S-Y Ning Y-L Wang H Peng Y. Perivascular AQP4 dysregulation in the hippocampal CA1 area after traumatic brain injury is alleviated by adenosine A(2A) receptor inactivation. Sci Rep. (2017) 7:2254. 10.1038/s41598-017-02505-6 28533515 PMC5440401

[176] Corrigan F Mander KA Leonard AV Vink R. Neurogenic inflammation after traumatic brain injury and its potentiation of classical inflammation. J Neuroinflammation. (2016) 13:264. 10.1186/s12974-016-0738-9 27724914 PMC5057243

[177] Cork SC. The role of the vagus nerve in appetite control: implications for the pathogenesis of obesity. J Neuroendocrinol. (2018) 30:e12643. 10.1111/jne.12643 30203877

[178] O'Leary OF Ogbonnaya ES Felice D Levone BR Conroy LC Fitzgerald P. The vagus nerve modulates BDNF expression and neurogenesis in the hippocampus. Eur Neuropsychopharmacol. (2018) 28:307–16. 10.1016/j.euroneuro.2017.12.004 29426666

[179] Frago L Baquedano E Argente J Chowen J. Neuroprotective actions of ghrelin and growth hormone secretagogues. Front Mol Neurosci. (2011) 4:23. 10.3389/fnmol.2011.00023 21994488 PMC3182030

[180] Qi L Cui X Dong W Barrera R Nicastro J Coppa GF. Ghrelin attenuates brain injury after traumatic brain injury and uncontrolled hemorrhagic shock in rats. Mol Med. (2012) 18:186–93. 10.2119/molmed.00390 22160303 PMC3320141

[181] Spencer SJ Miller AA Andrews ZB. The role of ghrelin in neuroprotection after ischemic brain injury. Brain Sci. (2013) 3:344–59. 10.3390/brainsci3010344 24961317 PMC4061836

[182] Cheng Y Chen B Xie W Chen Z Yang G Cai Y. Ghrelin attenuates secondary brain injury following intracerebral hemorrhage by inhibiting NLRP3 inflammasome activation and promoting Nrf2/ARE signaling pathway in mice. Int Immunopharmacol. (2020) 79:106180. 10.1016/j.intimp.2019.106180 31926478

[183] Postolache TT Wadhawan A Can A Lowry CA Woodbury M Makkar H. Inflammation in traumatic brain injury. J Alzheimers Dis. (2020) 74:1–28. 10.3233/JAD-191150 32176646 PMC8190673

[184] Bansal V Costantini T Ryu SY Peterson C Loomis W Putnam J. Stimulating the central nervous system to prevent intestinal dysfunction after traumatic brain injury. J Trauma. (2010) 68:1059–64. 10.1097/TA.0b013e3181d87373 20453760 PMC4251579

[185] Bansal V Ryu SY Lopez N Allexan S Krzyzaniak M Eliceiri B. Vagal stimulation modulates inflammation through a ghrelin mediated mechanism in traumatic brain injury. Inflammation. (2012) 35:214–20. 10.1007/s10753-011-9307-7 21360048 PMC3282000

[186] Engineer ND Kimberley TJ Prudente CN Dawson J Tarver WB Hays SA. Targeted Vagus Nerve Stimulation for Rehabilitation After Stroke. Front Neurosci. (2019) 13:280. 10.3389/fnins.2019.00280 30983963 PMC6449801

[187] Pruitt DT Schmid AN Kim LJ Abe CM Trieu JL Choua C. Vagus nerve stimulation delivered with motor training enhances recovery of function after traumatic brain injury. J Neurotrauma. (2016) 33:871–9. 10.1089/neu.2015.3972 26058501 PMC4860663

[188] Meyers EC Solorzano BR James J Ganzer PD Lai ES Rennaker RL 2nd. Vagus nerve stimulation enhances stable plasticity and generalization of stroke recovery. Stroke. (2018) 49:710–7. 10.1161/STROKEAHA.117.019202 29371435 PMC6454573

[189] Dawson J Liu CY Francisco GE Cramer SC Wolf SL Dixit A. Vagus nerve stimulation paired with rehabilitation for upper limb motor function after ischaemic stroke (VNS-REHAB): a randomised, blinded, pivotal, device trial. Lancet. (2021) 397:1545–53. 10.1016/S0140-6736(21)00475-X 33894832 PMC8862193

[190] Hulsey DR Shedd CM Sarker SF Kilgard MP Hays SA. Norepinephrine and serotonin are required for vagus nerve stimulation directed cortical plasticity. Exp Neurol. (2019) 320:112975. 10.1016/j.expneurol.2019.112975 31181199 PMC6708444

[191] Srihagulang C Vongsfak J Vaniyapong T Chattipakorn N Chattipakorn SC. Potential roles of vagus nerve stimulation on traumatic brain injury: evidence from *in vivo* and clinical studies. Exp Neurol. (2022) 347:113887. 10.1016/j.expneurol.2021.113887 34624329

[192] Oostra E Jazdzyk P Vis V Dalhuisen I Hoogendoorn A Planting C. More rTMS pulses or more sessions? The impact on treatment outcome for treatment resistant depression. Acta Psychiatr Scand. (2025) 151:485–505. 10.1111/acps.13768 39569643 PMC11884915

[193] Jassam YN Izzy S Whalen M McGavern DB El Khoury J. Neuroimmunology of traumatic brain injury: time for a paradigm shift. Neuron. (2017) 95:1246–65. 10.1016/j.neuron.2017.07.010 28910616 PMC5678753

[194] Mokbel AY Burns MP Main BS. The contribution of the meningeal immune interface to neuroinflammation in traumatic brain injury. J Neuroinflammation. (2024) 21:135. 10.1186/s12974-024-03122-7 38802931 PMC11131220

[195] Bolte AC Shapiro DA Dutta AB Ma WF Bruch KR Kovacs MA. The meningeal transcriptional response to traumatic brain injury and aging. Elife. (2023) 12:e81154. 10.7554/eLife.81154 36594818 PMC9810333

[196] Bolte AC Dutta AB Hurt ME Smirnov I Kovacs MA McKee CA. Meningeal lymphatic dysfunction exacerbates traumatic brain injury pathogenesis. Nat Commun. (2020) 11:4524. 10.1038/s41467-020-18113-4 32913280 PMC7483525

[197] Buenaventura RG Harvey AC Burns MP Main BS. Traumatic brain injury induces an adaptive immune response in the meningeal transcriptome that is amplified by aging. Front Neurosci. (2023) 17:1210175. 10.3389/fnins.2023.1210175 37588516 PMC10425597

[198] Liao S Liu T Yang R Tan W Gu J Deng M. Structure and function of sodium channel Nav1.3 in neurological disorders. Cell Mol Neurobiol. (2023) 43:575–84. 10.1007/s10571-022-01211-w 35332400 PMC11415190

[199] Menezes LFS Sabiá Júnior EF Tibery DV Carneiro LDA Schwartz EF. Epilepsy-related voltage-gated sodium channelopathies: a review. Front Pharmacol. (2020) 11:1276. 10.3389/fphar.2020.01276 33013363 PMC7461817

[200] Liu H Hu J Zheng Q Feng X Zhan F Wang X. Piezo1 channels as force sensors in mechanical force-related chronic inflammation. Front Immunol. (2022) 13:816149. 10.3389/fimmu.2022.816149 35154133 PMC8826255

[201] Liang J Huang B Yuan G Chen Y Liang F Zeng H. Stretch-activated channel Piezo1 is up-regulated in failure heart and cardiomyocyte stimulated by AngII. Am J Transl Res. (2017) 9:2945–55. 28670382 PMC5489894

[202] Tuszynski MH. Growth-factor gene therapy for neurodegenerative disorders. Lancet Neurol. (2002) 1:51–7. 10.1016/S1474-4422(02)00006-6 12849545

